# Commercial Chinese polyherbal preparation Zao Ren An Shen prescription for primary insomnia: a systematic review with meta-analysis and trial sequential analysis

**DOI:** 10.3389/fphar.2024.1376637

**Published:** 2024-06-18

**Authors:** Fei-Yi Zhao, Peijie Xu, Gerard A. Kennedy, Zhen Zheng, Wen-Jing Zhang, Jia-Yi Zhu, Yuen-Shan Ho, Li-Ping Yue, Qiang-Qiang Fu, Russell Conduit

**Affiliations:** ^1^ Department of Nursing, School of International Medical Technology, Shanghai Sanda University, Shanghai, China; ^2^ Shanghai Municipal Hospital of Traditional Chinese Medicine, Shanghai University of Traditional Chinese Medicine, Shanghai, China; ^3^ School of Computing Technologies, RMIT University, Melbourne, VIC, Australia; ^4^ Institute of Health and Wellbeing, Federation University, Ballarat, VIC, Australia; ^5^ School of Health and Biomedical Sciences, RMIT University, Melbourne, VIC, Australia; ^6^ Institute for Breathing and Sleep, Austin Health, Heidelberg, VIC, Australia; ^7^ Yangpu Hospital, School of Medicine, Tongji University, Shanghai, China; ^8^ School of Nursing, Faculty of Health and Social Sciences, The Hong Kong Polytechnic University, Kowloon, China

**Keywords:** Zao Ren An Shen, insomnia, sleep quality, botanical drugs, Chinese medicine, systematic review, meta-analysis, clinical trials

## Abstract

**Background:** Natural products are widely used for primary insomnia (PI). This systematic review with trial sequential analysis (TSA) aimed to summarize evidence pertaining to the effectiveness and safety of Zao Ren An Shen (ZRAS) prescription, a commercial Chinese polyherbal preparation, for treating PI.

**Methods:** Controlled clinical trials appraising ZRAS compared to controls or as an add-on treatment were systematically searched across seven databases until January 2024. Cochrane ROB 2.0 and ROBINS-I tools were adopted to determine risk of bias. Quality of evidence was assessed using the GRADE framework.

**Results:** We analyzed 22 studies, involving 2,142 participants. The effect of ZRAS in reducing Pittsburgh Sleep Quality Index scores was found to be comparable to benzodiazepines [*MD* = 0.39, 95%*CI* (−0.12, 0.91), *p* = 0.13] and superior to Z-drugs [*MD* = −1.31, 95%*CI* (−2.37, −0.24), *p* = 0.02]. The addition of ZRAS to hypnotics more significantly reduced polysomnographically-recorded sleep onset latency [*MD* = −4.44 min, 95%*CI* (−7.98, −0.91), *p* = 0.01] and number of awakenings [*MD* = −0.89 times, 95%*CI* (−1.67, −0.10), *p* = 0.03], and increased total sleep time [*MD* = 40.72 min, 95%*CI* (25.14, 56.30), *p* < 0.01], with fewer adverse events than hypnotics alone. TSA validated the robustness of these quantitative synthesis results. However, the quality of evidence ranged from very low to low. The limited data available for follow-up did not support meta-synthesis.

**Conclusion:** While ZRAS prescription shows promising effectiveness in treating PI, the overall quality of evidence is limited. Rigorously-designed randomized control trials are warranted to confirm the short-term efficacy of ZRAS and explore its medium-to-long-term efficacy.

**Systematic Review Registration:** (https://www.crd.york.ac.uk/prospero/display_record.php?RecordID=471497), identifier (CRD42023471497).

## Graphical Abstract

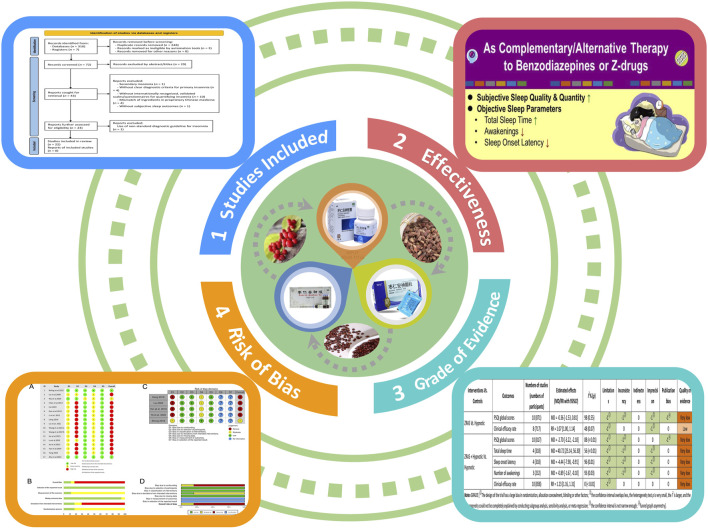

## 1 Background

Approximately 30% of the general population worldwide suffers from chronic insomnia (Roth, 2007), with primary insomnia (PI) accounting for an estimated 25% of these cases (Roth and Roehrs, 2003). PI manifests as frequent sleep disturbances, where the subjective complaint of trouble falling asleep, maintaining sleep or non-restorative sleep, or the experience of sleep that is non-refreshing, cannot be directly attributed to a comorbid psychiatric and/or medical disorder (Shekleton et al., 2010). Insufficient sleep duration has been linked to a variety of deleterious health outcomes, cardiovascular disease, diabetes, weight gain and obesity (Grandner et al., 2016), heightened susceptibility to anxiety and depressive disorders (Neckelmann et al., 2007), and increased mortality (Grandner et al., 2016). Insomnia also imparts tremendous societal and economic ramifications, resulting from workplace absenteeism, reduced productivity, and increased accident rates (Zhou et al., 2017). The combined direct and indirect healthcare costs associated with insomnia have been estimated at up to $100 billion annually (Taddei-Allen, 2020).

A proportion of patients worldwide living with insomnia resort to various types of complementary and alternative medicine (CAM) treatments (Xie et al., 2013; Zhao et al., 2021), including Chinese medicines (Birling et al., 2019; Birling et al., 2022). Commercial Chinese polyherbal preparation (CCPP) refers to Chinese medicinal products, used under the guidance of the traditional Chinese medicine (TCM) theory, that have been manufactured into solution, pill, powder, ointment, capsule, tablet, concentrated extract granules, etc. (Chao et al., 2021).

Zao Ren An Shen (ZRAS; Chinese character: 棗仁安神) is a CCPP composed of three botanical drugs: Ziziphi spinosae semen, Schisandrae chinensis fructus and Salviae miltiorrhizae radix et rhizoma (Birling et al., 2020; Birling et al., 2022) (Table 1). Ziziphi spinosae semen refers to the dried seed of *Ziziphus jujuba* Mill. var. *Spinosa* (Bunge) Hu ex H. F. Chou (Zhou et al., 2018). Schisandrae chinensis fructus is the dry ripe fruits of *Schisandra chinensis* (Turcz.) Baill. (Yang et al., 2022). Salviae miltiorrhizae radix et rhizoma refers to the dried root of *Salvia miltiorrhiza* Bunge (Wang et al., 2017). Despite being a modern Chinese medicinal preparation, the three botanical drugs in ZRAS have been used in TCM for mental illness for thousands of years. All these three were first documented in the ancient TCM monograph *Shennong’s Classic of Materia Medica* (SCMM) over 2500 years ago (Wang et al., 2017; Zhou et al., 2018; Yang et al., 2022). Ziziphi spinosae semen is annotated in the SCMM for its efficacy in calming the *Heart* and tranquilizing the *Mind* and is recommended for treating restlessness, palpitations, insomnia and excessive dreaming (Guan et al., 2022). It is also the most commonly used single botanical drug for treating insomnia in TCM (Zhou et al., 2018). The SCMM records the efficacy of Schisandrae chinensis fructus in nourishing the *Kidney* and calming the *Mind*, and it is therefore often used to treat insomnia and depression (Yang et al., 2022). Salviae miltiorrhizae radix et rhizoma is used to treat insomnia due to its documented efficacy in clearing the *Heart* and eliminating restlessness, as noted in the SCMM (Wang et al., 2017).

**TABLE 1:** The information about the botanical drugs contained in ZRAS.

Botanical drug	Plant	Family
Ziziphi spinosae semen	*Ziziphus jujuba* Mill. var. *Spinosa* (Bunge) Hu ex H. F. Chou	Rhamnaceae
Schisandrae chinensis fructus	*Schisandra chinensis* (Turcz.) Baill	Schisandraceae
Salviae miltiorrhizae radix et rhizoma	*Salvia miltiorrhiza* Bunge	Lamiaceae

ZRAS is manufactured as CCPP in the form of granules, capsules, or solution, with the former two included in the *Pharmacopoeia of the People*’*s Republic of China* (PPRC), 2020 version (https://ydz.chp.org.cn/#/main) for insomnia treatment. The prescription, preparation methods, characteristics, therapeutic functions, dosage and administration, specifications, precautions, and storage method of ZRAS capsules [Sinopharm Group Tongjitang (Guizhou) Pharmaceuticals Co., Ltd.; SFDA Approval No. Z20010033)] and ZRAS granules (Heilongjiang province Jiren Pharmaceuticals Co., Ltd.; SFDA Approval No. Z20053837) are comprehensively elucidated in the PPRC, as presented in Supplementary Appendix S1.

Chemical analysis suggests that jujubosides, schisandrin, triterpene saponin glycosides, flavonoids, and alkaloids are present in Ziziphi spinosae semen, Schisandrae chinensis fructus and/or Salviae miltiorrhizae radix et rhizoma, which may account for the biological effects of ZRAS (Birling et al., 2020). These metabolites regulate sleep-wake cycles by either enhancing the metabolism of gamma-aminobutyric acid or modulating the serotonergic systems (Birling et al., 2020).

ZRAS is the most extensively used and researched CCPP for insomnia (Birling et al., 2020; Birling et al., 2022; Yu et al., 2022). However, a double-blind randomized control trial (RCT) from Australia found that ZRAS capsule to be no more effective than placebo for insomnia (Birling et al., 2022), contrasting with findings from two similar RCTs conducted in China (Liu and Nan, 2009; Wu and Jiang, 2020). These inconsistent findings promoted us to conduct a systematic review. Two systematic reviews (Birling et al., 2020; Chen et al., 2020) published in peer-reviewed journals earlier covered similar topics but did not specifically focus on PI. Instead, they combined evidence on both PI and secondary insomnia. This approach introduces additional variability in quantitative synthesis, potentially skewing the actual therapeutic efficacy evaluation of ZRAS and complicating the identification of its specific indications. Moreover, these reviews were conducted in 2018 (Birling et al., 2020) and 2019 (Chen et al., 2020) respectively. New evidence may be available. Our study exclusively focused on PI, incorporating recent clinical evidence to minimize bias and obtain more objective conclusions. Additionally, we utilized trial sequential analysis (TSA) to appraise the robustness of quantitative synthesis results (Sanfilippo et al., 2021), a component lacking in previous reviews.

By combining all available data, we aimed to objectively assess the role and safety of ZARS in PI treatment and thus, enable clinicians to make critically-evaluated evidence-based treatment decisions about its use.

## 2 Materials and methods

### 2.1 Registration

The current review followed the *Preferred Reporting Items for Systematic Reviews and Meta-Analyses (PRISMA) 2020 Statement* guidelines (Page et al., 2021). A prospective protocol was registered in PROSPERO (Identifier: CRD42023471497).

### 2.2 Eligibility criteria

#### 2.2.1 Inclusion criteria based on the PICOS framework

In line with the PICOS framework, formally published RCTs and non-randomized controlled clinical trials (NRCTs) were included, irrespective of language and date restrictions. Patients must be diagnosed with insomnia as per standard diagnostic criteria (Suppelmentary Appendix S2.1). Interventions were restricted to ZRAS, either as a standalone treatment or in combination with standard care for PI, i.e., hypnotics/sedatives, or cognitive behavioral therapy for insomnia (CBT-i). Controls included waitlist-control, placebo-ZRAS, or standard care. Placebo-hypnotic combined with ZRAS was only allowed as an eligible intervention when the same hypnotics combined with placebo-ZRAS was applied to controls. Primary outcomes were restricted to internationally recognized, validated scales/questionnaires for quantifying insomnia, i.e., Pittsburgh Sleep Quality Index (PSQI), Insomnia Severity Index (ISI) and Athens Insomnia Scale (AIS).

#### 2.2.2 Exclusion criteria for the study

To ensure the reliability of the results, studies adopting only self-developed sleep questionnaires were not considered. Secondary outcomes included sleep diary, objective sleep parameters measured with actigraphy or polysomnography, clinical efficacy rate, and adverse events (AEs). The studies were also excluded if they: 1) only included objective sleep outcomes without subjective measures; 2) employed ZRAS as a decoction rather than a CCPP; and/or 3) used compounded ZRAS. This review does not discuss compounded ZARS products due to the disparity in drug ingredients. For instance, an in-hospital medication preparation called “Compounded ZRAS Capsule” (SFDA Approval No. Z20080002), manufactured by the First Affiliated Hospital of Anhui University of Chinese Medicine, consists of six ingredients: Ziziphi spinosae semen, Platycladi semen, Aucklandiae radix, Polygalae radix, Angelicae sinensis radix and Coptidis rhizoma (Xu et al., 2015). Another compounded product, “Quick-Acting ZRAS Capsule,” manufactured by Chongqing Pharmaceutical Factory of Chinese Medicine (SFDA Approval No. 008351), mainly comprises Ziziphi spinosae semen and *L*-Tetrahydropalmatine (Luo et al., 1999).

### 2.3 Search strategy and data extraction

A systematic search was conducted across seven electronic databases [Cochrane Central Register of Controlled Trials (CENTRAL), MEDLINE (via PubMed), EMBASE, China biomedical literature service system (SinoMed), China National Knowledge Infrastructure (CNKI), Chongqing VIP database (CQVIP) and Wanfang database and two trial registries [WHO International clinical trials registry platform search portal and US ClinicalTrials.gov] from their inceptions through January 2024. The search strategies with search terms were detailed in Supplementary Appendix S2.2.

Search outcomes were exported to *Rayyan* for duplicate removal. Initial screening of titles and abstracts facilitated the exclusion of obviously irrelevant studies, with full texts reviewed when necessary. Two independent reviewers selected eligible trials, achieving consensus on inclusion. A spreadsheet was utilized for data extraction, encompassing trial identification details, participant grouping specifics (number of patients, gender ratio per group, duration of PI, patients’ TCM syndrome patterns), diagnostic criteria, intervention protocols (e.g., dosage form, dose, frequency, etc.), prescription in controls, outcome measures, results, follow-up details, and AEs.

### 2.4 Evaluation of risk of bias in individual studies

Two investigators (W-JZ and JY-Z) independently appraised the included trials. A substantial level of agreement (*Kappa* = 0.86) was achieved, and all discrepancies were solved by discussion followed by consensus and arbitrated by a third assessor (FY-Z). The methodological quality of the RCTs and NRCTs were appraised using Revised Cochrane Risk of Bias tool for randomised trials (ROB 2.0) (Sterne et al., 2019) and Cochrane Risk of Bias in Non-randomized Studies-of Interventions (ROBINS-I) (Sterne et al., 2016), respectively.

## 3 Data analysis

We analyzed all studies qualitatively. Outcomes measured in at least three trials were merged for quantitative meta-analysis using Review Manager 5.4.1 software. Continuous variables (i.e., sleep scales/questionnaires scores and objective sleep parameters) were pooled using the inverse variance method, while dichotomous variables (i.e., clinical efficacy rate) were pooled using the Mantel-Haenszel method. Statistical heterogeneity was appraised using the *Chi* ^*2*^ test and was quantified by *I* ^*2*^ statistic. Following the recommendations of Tufanaru *et al*. (Tufanaru et al., 2015), the random-effects model was employed as the default model, as we aimed to generalize the conclusions beyond the actual studies included in the meta-analysis. The fixed-effects model was only considered in cases where there was no statistical heterogeneity among the effect sizes (*I* ^*2*^ = 0), or when the number of pooled studies was less than five and the heterogeneity was acceptable, i.e., *p* > 0.10 and *I* ^*2*^ ≤ 50%. We adopted TSA for primary outcomes, assessing whether the sample sizes were adequate enough to generate a statistically significant result. TSA was performed with a two-tailed type I error rate of 5% and 80% power using TSA 0.9.5.10 Beta software.

In case where there was significant clinical heterogeneity and the data permitted, subgroup analyses were performed based on different study types (RCT or NRCT), dosage forms of ZRAS, therapeutic dosage (high dosage with treatment ≥4 weeks; low dosage with treatment <4 weeks), comparator interventions (pharmacotherapy or psychotherapy), and medications in the controls (benzodiazepines, Z-drugs, melatonin receptor agonist, dual orexin receptor antagonist, or sedative antidepressants). We also conducted meta-regression analysis and sensitivity analysis if sufficient studies were available (*n* ≥ 10). Univariate or multifactor meta-regressions were carried out by taking publication year, study sample size, study types, dosage forms of ZRAS, therapeutic dosage and/or medications in the controls as covariates if data permitted. In addition to identifying the sources of heterogeneity, sensitivity analysis with influence analysis method was also used to check robustness of the conclusions derived from meta-synthesis.

With STATA 18.0 software, we employed linear regression analysis (*Egger’s* test) to detect the potential publication bias for outcomes measured in at least ten trials.

## 4 Assessment on the certainty of evidence

The overall quality of evidence obtained from the meta-synthesis was appraised using the Grades of Recommendation, Assessment, Development, and Evaluation (GRADE) framework (Atkins et al., 2004). The certainty of evidence was categorized into four levels, ranging from “High” to “Very low.” The GRADE approach classifies bodies of RCTs as initially starting at high certainty and bodies of non-randomized studies at initially starting at low certainty (Schwingshackl et al., 2021). Accordingly, evidence bodies studied exclusively in RCTs were initially assigned *a priori* ranking of “High,” whereas other evidence bodies were initially assigned the ranking of “Low.” Subsequently, evidence bodies might undergo further upgrading when multiple high-quality studies yield consistent results or downgrading due to identifiable bias (Goldet and Howick, 2013).

## 5 Results analysis

**FIGURE 1:**
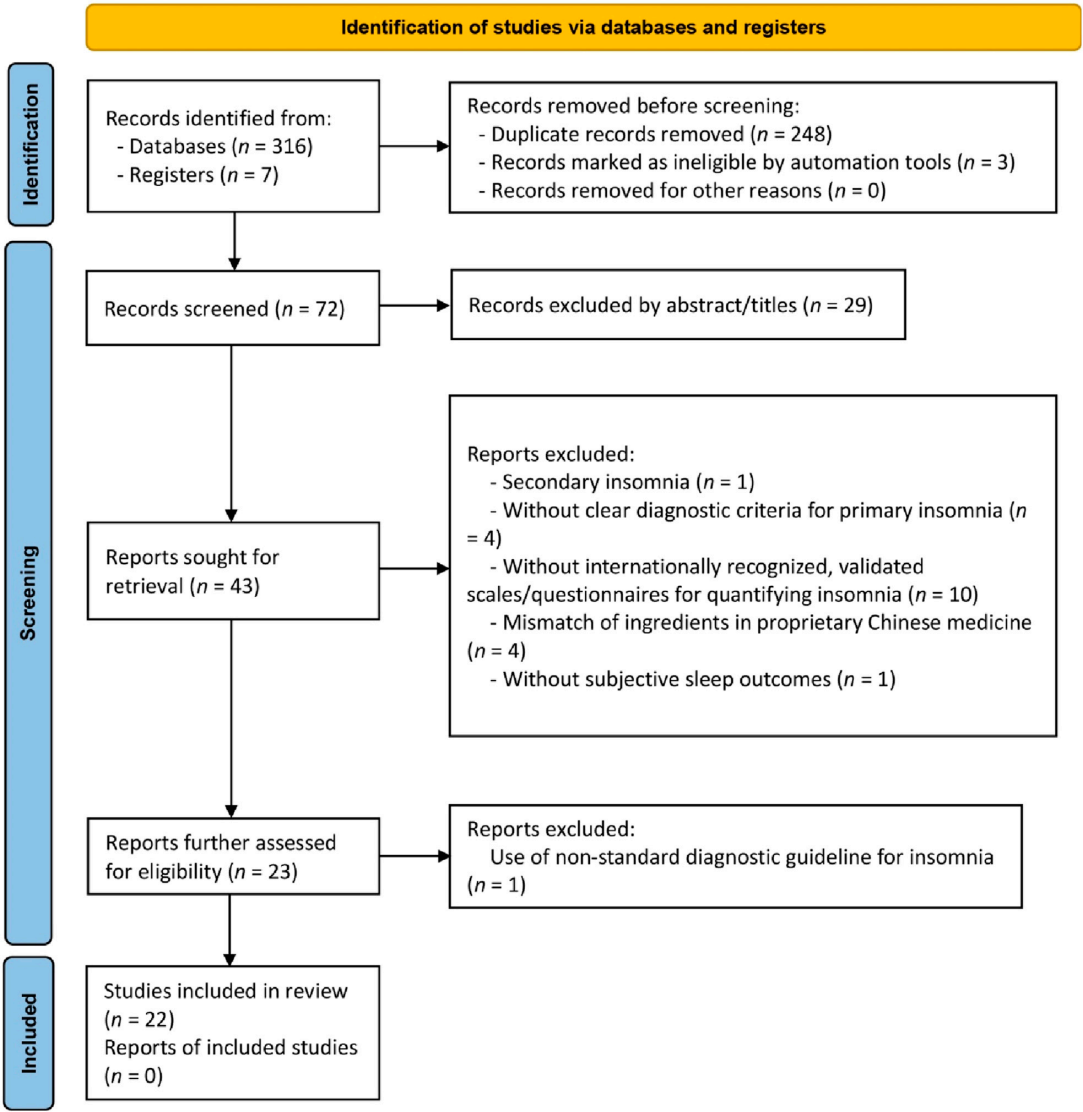
Flow diagram of the study selection process.

The initial search yielded 323 articles. Following duplicate removal and thorough full-text screening, 17 RCTs and five NRCTs, composing a total of 2,142 participants from these 22 studies, met the predefined criteria (Figure 1). A summary of the discarded studies with specific reasons for irrelevance is provided in Supplementary Appendix S3.

### 5.1 Description of studies

Among the 22 included trials (Table 2), three compared ZRAS against placebo-ZRAS, eight against hypnotics alone, and 11 evaluated ZRAS combined with hypnotics versus hypnotics alone. None of these trials included CBT-i or waitlist-control. The PPRC-recommended dose for ZRAS capsule or granule was consistently administered across all trials. A variety of hypnotics were used as active controls, with Estazolam (9/20), Eszopiclone (4/20), Zolpidem (3/20), Zopiclone (2/20), Alprazolam (1/20) and Oxazepam (1/20), in descending order of frequency of use.

**TABLE 2:** Characteristics of the included studies.

Author, year	Study type	Group/size (M = male, F = female)	Age (year)	Disease duration (d = day; m = month; y = year)	Diagnostic system	TCM syndrome Pattern	ZRAS interventions	Dosage form of ZRAS	Prescription in control group (placebo /Western medication)	Outcome measure tool	ZRAS/ZRAS + Hypnotics/Sedatives compared with controls (waitlist, placebo-ZRAS, Hypnotics/Sedatives, or CBT-i)	Follow-up	Adverse events
Birling *et al.* 2022 (Birling et al., 2022)	RCT	- ZRAS/n = 38 (9M, 29F) - placebo-ZRAS/n = 47 (17M, 30F)	- ZRAS/52.0 ± 13.0 - placebo-ZRAS/50.0 ± 16.0	- ZRAS/NR - placebo-ZRAS/NR	DSM-5	NR	0.76g*3^#^ qn for 4 weeks	capsule	placebo-ZRAS 0.72g*3^#^ qn for 4 weeks	(i) ISI (ii) DASS (iii) FSS (iv) AQoL (v-i) sleep diary [TST (min)] (v-ii) sleep diary [SOL (min)] (v-iii) sleep diary [WASO (min)] (vi-i) actigraphy [TST (min)] (vi-ii) actigraphy [SOL (min)] (vi-iii) actigraphy [WASO (min)]	(i) compared with placebo-ZRAS *p* > 0.05 (ii) compared with placebo-ZRAS *p* > 0.05 (iii) compared with placebo-ZRAS *p* > 0.05 (iv) compared with placebo-ZRAS *p* > 0.05 (v-i) compared with placebo-ZRAS *p* > 0.05 (v-ii) compared with placebo-ZRAS *p* > 0.05 (v-iii) compared with placebo-ZRAS *p* > 0.05 (vi-i) compared with placebo-ZRAS *p* > 0.05 (vi-ii) compared with placebo-ZRAS *p* > 0.05 (vi-iii) compared with placebo-ZRAS *p* > 0.05	4-week follow-up (no significant differences in efficacy outcomes between two groups)	- ZRAS/n = 10 [dry mouth (2); frequent night waking (1); facial skin rash (1); urinary urgency (n = 1); NR (5)] - placebo-ZRAS/n = 16 [NR]
Liu *et al.* 2009 (Liu and Nan, 2009)	RCT	- ZRAS/n = 30 (12M, 18F) - placebo-ZRAS/n = 30 (13M, 17F)	- ZRAS/36.9 ± 11.5 - placebo-ZRAS/37.8 ± 11.4	- ZRAS/8.9 ± 2.8m - placebo-ZRAS/9.8 ± 2.3m	ICSD-3	NR	0.45g*5^#^ qn for 3 weeks	capsule	placebo-ZRAS 0.45g*5^#^ qn for 3 weeks	(i) PSQI (ii) clinical efficacy rate	(i) compared with placebo-ZRAS *p* < 0.05 (ii) compared with placebo-ZRAS *p* < 0.05	no follow-up	NR
Liu *et al.* 2009 (Liu and Nan, 2009)	RCT	- ZRAS/n = 30 (12M, 18F) - Estazolam/n = 30 (13M, 17F)	- ZRAS/36.9 ± 11.5 - Estazolam/37.2 ± 11.4	- ZRAS/8.9 ± 2.8m - Estazolam/9.3 ± 2.2m	ICSD-3	NR	0.45g*5^#^ qn for 3 weeks	capsule	Estazolam 1mg qn for 3 weeks	(i) PSQI (ii) clinical efficacy rate	(i) compared with Estazolam *p* > 0.05 (ii) compared with Estazolam *p* > 0.05	no follow-up	NR
Wu *et al.* 2020 (Wu and Jiang, 2020)	RCT	- ZRAS/n = 120 (48M, 72F) - placebo-ZRAS/n = 120 (45M, 75F)	- ZRAS/51.1 ± 8.3 - placebo-ZRAS/50.9 ± 9.0	- ZRAS/12.1 ± 3.5m - placebo-ZRAS/12.1±4.1m	DSM-5	NR	0.45g*5^#^ qn for 4 weeks	capsule	placebo-ZRAS 0.45g*5^#^ qn for 4 weeks	(i) PSQI (ii) clinical efficacy rate	(i) compared with placebo-ZRAS *p* < 0.05 (ii) compared with placebo-ZRAS *p* < 0.01	no follow-up	- ZRAS/n = 14 [fatigue (2); dizziness (4); drowsiness (1); gastrointestinal discomfort (2); muscle soreness (1); dry mouth (2); constipation (2)] - placebo-ZRAS/n = 12 [fatigue (4); dizziness (2); drowsiness (1); gastrointestinal discomfort (2); muscle soreness (1); dry mouth (1); constipation (1)]
Zhu *et al.* 2022 (Zhu et al., 2022)	RCT	- ZRAS + placebo-Zolpidem/n = 32 (11M, 21F) - placebo-ZRAS + Zolpidem/n = 35 (10M, 25F)	- ZRAS + placebo-Zolpidem/51.0 ± 13.0 - placebo-ZRAS + Zolpidem/44.0 ± 13.0	NR	DSM-5	NR	0.45g*5^#^ for 4 weeks	capsule	Zolpidem 5mg + placebo-ZRAS qn for 4 weeks	(i) ISI (ii) clinical efficacy rate	(i) compared with placebo-ZRAS + Zolpidem *p* > 0.05 (ii) compared with placebo-ZRAS + Zolpidem *p* > 0.05	2-week follow-up (no significant differences in efficacy outcomes between two groups)	- ZRAS + placebo-Zolpidem/n = 2 [constipation (1); abdominal pain (1)] - placebo-ZRAS + Zolpidem/n = 6 [drowsiness (2); dry mouth/bitter taste (1); constipation and urinary urgency (1); gastrointestinal discomfort (1); head painful distension (1)]
Zhu *et al.* 2022 (Zhu et al., 2022)	RCT	- ZRAS + Zolpidem/n = 32 (10M, 22F) - placebo-ZRAS + Zolpidem/n = 35 (10M, 25F)	- ZRAS + Zolpidem/48.0 ± 12.0 - placebo-ZRAS + Zolpidem/44.0 ± 13.0	NR	DSM-5	NR	0.45g*5^#^ for 4 weeks	capsule	Zolpidem 5mg + placebo-ZRAS qn for 4 weeks	(i) ISI (ii) clinical efficacy rate	(i) compared with placebo-ZRAS + Zolpidem *p* < 0.05 (ii) compared with placebo-ZRAS + Zolpidem *p* < 0.05	2-week follow-up (lower ISI in ZRAS + Zolpidem group)	- ZRAS + Zolpidem/n = 3 [drowsiness (1); dry mouth (1); dizziness (1)] - placebo-ZRAS + Zolpidem/n = 6 [drowsiness (2); dry mouth/bitter taste (1); constipation and urinary urgency (1); gastrointestinal discomfort (1); head painful distension (1)]
Kang 2019 (Kang, 2019)	NRCT	- ZRAS + Zolpidem/n = 43 (22M, 21F) - Zolpidem/n = 43 (24M, 19F)	- ZRAS + Zolpidem/43.9 ± 1.8 - Zolpidem/43.6 ± 1.5	- ZRAS + Zolpidem/1.9 ± 0.7y - Zolpidem/1.7 ± 0.5y	GDTICA	NR	0.45g*5^#^ for 4 weeks	capsule	Zolpidem 10mg qn for 4 weeks	(i) PSQI (ii-i) PSG [TST (min)] (ii-ii) PSG [SE (%)] (ii-iii) PSG [REM (min)] (ii-iv) PSG [SOL (min)] (ii-v) PSG [WASO (min)] (iii) HADS (iv) PHQ-9 (v) WEMWBS (vi) clinical efficacy rate (vii) serum 5-HT (viii) serum orexin-A (ix) serum cortisol (x) serum IL-2 (xi) serum IL-6	(i) compared with Zolpidem *p* < 0.05 (ii-i) compared with Zolpidem *p* < 0.05 (ii-ii) compared with Zolpidem *p* < 0.05 (ii-iii) compared with Zolpidem *p* < 0.05 (ii-iv) compared with Zolpidem *p* < 0.05 (ii-v) compared with Zolpidem *p* < 0.05 (iii) compared with Zolpidem *p* < 0.05 (iv) compared with Zolpidem *p* < 0.05 (v) compared with Zolpidem *p* < 0.05 (vi) compared with Zolpidem *p* < 0.05 (vii) compared with Zolpidem *p* < 0.05 (viii) compared with Zolpidem *p* < 0.05 (ix) compared with Zolpidem *p* < 0.05 (x) compared with Zolpidem *p* < 0.05 (xi) compared with Zolpidem *p* < 0.05	no follow-up	- ZRAS + Zolpidem/n = 0 - Zolpidem/n = 0
Xu *et al.* 2022 (Xu and Zhou, 2022)	RCT	ZRAS + Eszopiclone /n = 48 (34M, 14F) Eszopiclone /n = 48 (31M, 17F)	ZRAS + Eszopiclone /71.0 ± 6.7 Eszopiclone/71.4 ± 7.0	ZRAS + Eszopiclone /3.5 ± 0.4m Eszopiclone/3.7 ± 0.4m	GDTICA	NR	5g qn for 4 weeks	granule	Eszopiclone 2mg qn for 4 weeks	(i) PSQI (ii-i) PSG [SWS (min)] (ii-ii) PSG [SE (%)] (ii-iii) PSG [WASO (min)] (iii) serum BDNF (iv) serum NPY (v) serum TNF-α (vi) clinical efficacy rate	(i) compared with Eszopiclone *p* < 0.05 (ii-i) compared with Eszopiclone *p* < 0.05 (ii-ii) compared with Eszopiclone *p* < 0.05 (ii-iii) compared with Eszopiclone *p* < 0.05 (iii) compared with Eszopiclone *p* < 0.05 (iv) compared with Eszopiclone *p* < 0.05 (v) compared with Eszopiclone *p* < 0.05 (vi) compared with Eszopiclone *p* < 0.05	no follow-up	- ZRAS + Eszopiclone/n =6 [nausea (2); headache (1); fatigue (1); dizziness (2)] - Eszopiclone/n = 5 [nausea (1); headache (1); fatigue (2); dizziness (1)]
Yan *et al.* 2018 (Yan et al., 2018)	NRCT	- ZRAS + Estazolam/n = 35 (16M, 19F) - Estazolam/n = 35 (17M, 18F)	- ZRAS + Estazolam/46.2 ± 5.0 - Estazolam/48.1 ± 5.1	- ZRAS + Estazolam/57.0 ± 5.4d - Estazolam/58.0 ± 4.0d	ICD-10	NR	0.45g*5^#^ for 4 weeks	capsule	Estazolam 1-2mg qn for 4 weeks	(i) PSQI (ii-i) PSG [TST (min)] (ii-ii) PSG [SOL (min)] (ii-iii) PSG [ATs (times)] (iii) clinical efficacy rate	(i) compared with Estazolam *p* < 0.01 (ii-i) compared with Estazolam *p* < 0.01 (ii-ii) compared with Estazolam *p* < 0.01 (ii-iii) compared with Estazolam *p* < 0.05 (iii) compared with Estazolam *p* < 0.05	no follow-up	- ZRAS + Estazolam/n = 2 [fatigue (1); dizziness (1)] - Estazolam/n = 9 [drowsiness (4); dizziness (3); dry mouth (2)]
Yang 2023 (Yang, 2023)	RCT	- ZRAS + Estazolam/n = 30 (19M, 11F) - Estazolam/n = 30 (20M, 10F)	- ZRAS + Estazolam/39.6 ± 2.1 - Estazolam/39.4 ± 2.2	- ZRAS + Estazolam/11.3 ± 2.1m - Estazolam/11.2 ± 2.2m	GDTICA	deficiency of *Heart*-blood	5g qn for 4 weeks	granule	Estazolam 1-2mg qn for 4 weeks	(i) PSQI (ii-i) PSG [TST (min)] (ii-ii) PSG [SOL (min)] (ii-iii) PSG [ATs (times)] (iii) clinical efficacy rate	(i) compared with Estazolam *p* < 0.01 (ii-i) compared with Estazolam *p* < 0.05 (ii-ii) compared with Estazolam *p* < 0.05 (ii-iii) compared with Estazolam *p* < 0.05 (iii) compared with Estazolam *p* < 0.05	no follow-up	- ZRAS + Estazolam/n = 2 [dry mouth (1); dizziness (1)] - Estazolam/n = 2 [nausea (1); dry mouth (1)]
Ye *et al.* 2022 (Ye and Lin, 2022)	NRCT	ZRAS + Eszopiclone/n = 51 (24M, 27F) Eszopiclone/n = 51 (23M, 28F)	ZRAS + Eszopiclone /54.5 ± 10.7 Eszopiclone/54.1 ± 10.3	ZRAS + Eszopiclone /11.4 ± 0.9m Eszopiclone/11.3 ± 0.8m	CCMD-3	hyperactivity of *Fire* due to *Yin* deficiency	5g qn for 4 weeks	granule	Eszopiclone 2-3mg qn for 4 weeks	(i) PSQI (ii-i) PSG [TST (min)] (ii-ii) PSG [SOL (min)] (ii-iii) PSG [ATs (times)] (iii) clinical efficacy rate	(i) compared with Eszopiclone *p* < 0.05 (ii-i) compared with Eszopiclone *p* < 0.05 (ii-ii) compared with Eszopiclone *p* < 0.05 (ii-iii) compared with Eszopiclone *p* < 0.05 (iii) compared with Eszopiclone *p* < 0.05	no follow-up	- ZRAS + Eszopiclone/n = 2 [acid reflux (1); dry mouth (1)] - Eszopiclone/n = 10 [drowsiness (1); dry mouth (5); dizziness (4)]
Lu *et al.* 2023 (Lu et al., 2023)	RCT	- ZRAS + placebo-Zolpidem/n = 10 (5M, 5F) - placebo-ZRAS + Zolpidem/n = 17 (7M, 10F)	- ZRAS + placebo-Zolpidem/55.0 ± 13.4 - placebo-ZRAS + Zolpidem/49.5 ± 14.5	- ZRAS + placebo-Zolpidem/NR - placebo-ZRAS + Zolpidem/NR	ICSD-3	NR	0.45g*5^#^ for 4 weeks	capsule	Zolpidem 10mg qn + placebo-ZRAS 0.45g*5^#^ for 4 weeks	(i) PSQI (ii) ISI (iii) HAMA (iv) HAMD	(i) compared with placebo-ZRAS + Zolpidem *p* < 0.05 (ii-i) compared with placebo-ZRAS + Zolpidem *p* < 0.05 (iii) compared with placebo-ZRAS + Zolpidem *p* > 0.05 (iv) compared with placebo-ZRAS + Zolpidem *p* > 0.05	no follow-up	NR
Lu *et al.* 2023 (Lu et al., 2023)	RCT	- ZRAS + Zolpidem/n = 14 (6M, 8F) - placebo-ZRAS + Zolpidem/n = 12 (2M, 10F)	- ZRAS + Zolpidem/48.5 ± 11.7 - placebo-ZRAS + Zolpidem/46.0 ± 12.7	- ZRAS + Zolpidem/NR - placebo-ZRAS + Zolpidem/NR	ICSD-3	NR	0.45g*5^#^ for 4 weeks	capsule	Zolpidem 5mg qn for 4 weeks	(i) PSQI (ii) ISI (iii) HAMA (iv) HAMD	(i) compared with placebo-ZRAS + Zolpidem *p* < 0.05 (ii-i) compared with placebo-ZRAS + Zolpidem *p* < 0.05 (iii) compared with placebo-ZRAS + Zolpidem *p* > 0.05 (iv) compared with placebo-ZRAS + Zolpidem *p* > 0.05	no follow-up	NR
Zhong 2018 (Zhong, 2018)	NRCT	ZRAS + Oxazepam/n = 48 (NR) Oxazepam/n = 48 (NR)	42.4 ± 3.6	10.2 ± 1.5m	CCMD-3	NR	0.45g*5^#^ for 4 weeks	capsule	Oxazepam 15-30mg tid for 4 weeks	(i) PSQI (ii) clinical efficacy rate	(i) compared with placebo-ZRAS *p* < 0.05 (ii) compared with placebo-ZRAS *p* < 0.05	no follow-up	NR
Chen *et al.* 2014 (Chen et al., 2014)	RCT	- ZRAS/n = 60 (21M, 39F) - Zopiclone/n = 60 (20M, 40F)	- ZRAS/45.6 ± 12.1 - Zopiclone/45.2 ± 11.5	- ZRAS/13.9 ± 11.5m - Zopiclone/13.1 ± 11.9m	CCMD-3	deficiency of *Heart*-blood	5g qn for 4 weeks	granule	Zopiclone 7.5mg qn for 4 weeks	(i) PSQI (ii) clinical efficacy rate	(i) compared with Zopiclone *p* > 0.05 (ii) compared with Zopiclone *p* < 0.05	no follow-up	- ZRAS/n = 2 [acid reflux (1); mouth-numbing (1)] - Zopiclone/n = 16 [drowsiness (1); dry mouth (1); dizziness (4); headache (1); bitter taste (9)]
Cui 2019 (Cui, 2019)	RCT	- ZRAS/n = 43 (21M, 22F) - Zopiclone/n = 47 (25M, 22F)	- ZRAS/70.0 ± 6.0 - Zopiclone/70.0 ± 6.0	- ZRAS/1.2 ± 0.5y - Zopiclone/1.1 ± 0.5y	GDTICA	NR	0.45g*5 qn for 4 weeks	capsule	Zopiclone 3mg qn for 4 weeks	(i) PSQI	(i) compared with Zopiclone *p* > 0.05	no follow-up	- ZRAS/n = 2 [dry mouth (1); sweat (1)] - Zopiclone/n = 9 [constipation (1); dry mouth (3); headache (2); sweat (3)]
Gan *et al.* 2013 (Gan et al., 2013)	RCT	- ZRAS/n = 60 (23M, 37F) - Alprazolam/n = 60 (23M, 37F)	- ZRAS/67.2 ± 5.0 - Alprazolam/66.5 ± 9.2	- ZRAS/19.4 ± 6.1m - Alprazolam/20.6 ± 8.7m	CCMD-3	NR	0.45g*5^#^ qn for 4 weeks	capsule	Alprazolam 0.8mg qn for 4 weeks	(i) PSQI (ii) clinical efficacy rate	(i) compared with Alprazolam *p* > 0.05 (ii) compared with Alprazolam *p* > 0.05	no follow-up	- ZRAS/n = 8 [fatigue (6); diarrhea (2)] - Alprazolam/n = 32 [fatigue (12); dizziness (11); drowsiness (7); hangover effects (2)]
Li *et al.* 2012 (Li and Gong, 2012)	RCT	- ZRAS/n = 30 (13M, 17F) - Estazolam/n = 30 (15M, 15F)	- ZRAS/37.8 - Estazolam/37.4	- ZRAS/NR - Estazolam/NR	CCMD-2-R	NR	0.45g*5^#^ qn for 2 weeks	capsule	Estazolam 1 mg qn for 2 weeks	(i) PSQI (ii) clinical efficacy rate	(i) compared with Estazolam *p* > 0.05 (ii) compared with Estazolam *p* > 0.05	no follow-up	- ZRAS/n = 3 [fatigue] - Estazolam/n = 26 [fatigue (8); dizziness (10); drowsiness (5); hangover effects (3)]
Liang 2016 (Liang, 2016)	RCT	- ZRAS/n = 40 (NR) - Estazolam/n = 40 (NR)	68.1 ± 13.6	9.6 ± 2.2m	CCMD-3	NR	0.45g*5^#^ qn for 4 weeks	capsule	Estazolam 1 mg qn for 4 weeks	(i) PSQI (ii) clinical efficacy rate	(i) compared with Estazolam *p* > 0.05 (ii) compared with Estazolam *p* > 0.05	no follow-up	- ZRAS/n = 6 [fatigue (2); dizziness (1); drowsiness (3)] - Estazolam/n = 21 [fatigue (7); dizziness (6); drowsiness (8)]
Liu *et al.* 2021 (Liu and Gong, 2021)	RCT	- ZRAS/n = 52 (30M, 22F) - Eszopiclone/n = 52 (29M, 23F)	- ZRAS/48.3 ± 6.6 - Eszopiclone/48.6 ± 7.4	- ZRAS/9.5 ± 3.3m - Eszopiclone/9.4 ± 3.4m	ICSD-3	NR	0.45g*5^#^ for 4 weeks	capsule	Eszopiclone 3mg qn for 4 weeks	(i) PSQI (ii) GAD-7 (iii) FSS	(i) compared with Eszopiclone *p* < 0.05 (ii) compared with Eszopiclone *p* < 0.05 (iii) compared with Eszopiclone *p* < 0.05	no follow-up	- ZRAS /n =3 [muscle soreness (1); constipation (1); dizziness (1)] - Eszopiclone/n = 7 [muscle soreness (1); constipation (3); dizziness (1); nausea (1); dry mouth (1)]
Wang J. *et al.* 2017 (Wang J. et al., 2017)	RCT	- ZRAS/n = 52 (39M, 25F) - Eszopiclone/n = 64 (38M, 26F)	- ZRAS/42.5 ± 11.3 - Eszopiclone/42.6 ± 10.1	- ZRAS/10.1 ± 3.8m - Eszopiclone/10.3 ± 2.4m	GDTICA	NR	0.45g*5^#^ for 4 weeks	capsule	Eszopiclone 3mg qn for 4 weeks	(i) PSQI (ii) clinical efficacy rate	(i) compared with placebo-ZRAS *p* < 0.01 (ii) compared with placebo-ZRAS *p* < 0.01	no follow-up	- ZRAS /n = 2 [mouth-numbing (1); acid reflux (1)] - Eszopiclone/n = 9 [mouth-numbing (1); dizziness/headache (3); dry mouth/bitter taste (5)]
Wang X. *et al.* 2017 (Wang X. et al., 2017)	RCT	- ZRAS/n = 41 (20M, 21F) - Estazolam/n = 41 (19M, 22F)	- ZRAS/58.5 ± 5.2 - Estazolam/55.2 ± 5.3	- ZRAS/3.8 ± 0.9m - Estazolam/3.3 ± 0.5m	CCMD-3	NR	0.45g*5^#^ for 3 weeks	capsule	Estazolam 1mg qn for 3 weeks	(i) PSQI (ii) clinical efficacy rate	(i) compared with Estazolam *p* < 0.05 (ii) compared with Estazolam *p* < 0.05	no follow-up	- ZRAS/n = 5 [fatigue] - Estazolam/n = 23 [fatigue (11); dizziness (5); drowsiness (5); dry mouth (2)]
Hu *et al.* 2015 (Hu and Sheng, 2015)	RCT	- ZRAS + Estazolam/n = 50 (27M, 23F) - Estazolam/n = 43 (25M, 18F)	- ZRAS + Estazolam/49.8 ± 13.3 - Estazolam/45.3 ± 16.5	- ZRAS + Estazolam/16.4 ± 4.1m - Estazolam/15.1 ± 3.2m	ICD-10	NR	5g qn for 12 weeks	granule	Estazolam 1-2mg qn for 12 weeks	(i) PSQI (ii) clinical efficacy rate	(i) compared with Estazolam *p* < 0.05 (ii) compared with Estazolam *p* < 0.05	no follow-up	- ZRAS + Estazolam/n = 7 [fatigue (6); diarrhea (1)] - Estazolam/n = 20 [drowsiness (9); dizziness (7); drowsiness (4)]
Yan *et al.* 2019 (Yan et al., 2019)	RCT	- ZRAS + Estazolam/n = 39 (27M, 12F) - Estazolam/n = 39 (28M, 11F)	- ZRAS + Estazolam/39.6 ± 10.5 - Estazolam/39.4 ± 11.0	- ZRAS + Estazolam/12.3 ± 1.1m - Estazolam/11.9 ± 1.3m	GDTICA	NR	0.45g*5^#^ for 3 weeks	capsule	Estazolam 1mg qn for 3 weeks	(i) PSQI (ii) SCL-90 (iii) clinical efficacy rate	(i) compared with Estazolam *p* < 0.05 (ii) compared with Estazolam *p* < 0.05 (iii) compared with Estazolam *p* < 0.05	no follow-up	- ZRAS + Estazolam/n = 2 [drowsiness (1); fatigue (1)] - Estazolam/n = 8 [drowsiness (2); fatigue (1); dry mouth (1); dizziness (4)]
Liu 2022 (Liu, 2022)	NRCT	- ZRAS + Estazolam/n = 60 (29M, 31F) - Estazolam/n = 50 (28M, 22F)	- ZRAS + Estazolam/45.5 ± 10.2 - Estazolam/45.5 ± 10.2	- ZRAS + Estazolam/2.6 ± 0.7y - Estazolam/2.6 ± 0.7y	GDTICA	deficiency of *Heart*-blood	0.45g*5^#^ for 4 weeks	capsule	Estazolam 1mg qn for 4 weeks	(i) PSQI (ii) serum 5-HT (iii) serum GABA (iv) clinical efficacy rate	(i) compared with Estazolam *p* < 0.05 (ii) compared with Estazolam *p* < 0.05 (iii) compared with Estazolam *p* > 0.05 (iv) compared with Estazolam *p* < 0.05	no follow-up	- ZRAS + Estazolam/n =3 [fatigue (2); dizziness (1)] - Estazolam/n = 6 [drowsiness (3); fatigue (2); dry mouth (1)]

***Abbreviations*:** NR, no report; 5-HT, 5-hydroxytryptamine; AQoL, Assessment of Quality of Life; ATs, awakening times; BDNF, brain-derived neurotrophic factor; CCMD-2-R, Chinese Classification of Mental Disorders (Second Edition, Revised); CCMD-3, Chinese Classification of Mental Disorders (Third Edition); GDTICA, Guideline for the diagnosis and treatment of insomnia in Chinese adults; DASS, Depression Anxiety Stress Scales 21-item; DSM-V, Diagnostic and Statistical Manual of Mental Disorders (Fifth Edition); FSS, Fatigue Severity Scale; GABA, Gamma-amino butyric acid; GAD-7, 7-item Generalized Anxiety Disorder; HADS, Hospital Anxiety and Depression Scale; HAMA, Hamilton Anxiety Scale; HAMD, Hamilton Depression Scale; ICD-10, International Classification of Diseases (Tenth Revision); ICSD-3, International classification of sleep disorders (Third Edition); ISI, Insomnia Severity Index; LI-2, interleukin-2; LI-6, interleukin-6; NPY, neuropeptide Y; NRCT, non-randomized controlled clinical trial; PHQ-9, Patient Health Questionnaire-9; PSG, polysomnography; PSQI, Pittsburgh Sleep Quality Index; REM, rapid eye movement sleep; RCT, randomized control trial; SCL-90, Symptom Check List-90; SE, sleep efficiency; SOL, sleep onset latency; SWS, slow-wave sleep; TNF-α, tumor necrosis factor-α; TST, total sleep time; WASO, wake after sleep onset; WEMWBS, Warwick-Edinburgh Mental Wellbeing Scale; ZRAS, Zao Ren An Shen.

Two trials (Birling et al., 2022; Zhu et al., 2022) employed the ISI as the primary outcome measure in evaluating sleep quality and quantity, while others relied on PSQI. Six trials further recorded changes in objective sleep parameters using actigraphy (Birling et al., 2022) or polysomnography (Yan et al., 2018; Kang, 2019; Xu and Zhou, 2022; Ye and Lin, 2022; Yang, 2023). Sleep diary was adopted in only one trial (Birling et al., 2022). Additionally, 18 studies compared the clinical efficacy rate across interventions, albeit with varying grading criteria (Supplementary Appendix S4).

In all 22 included trials, the ZRAS capsules/granules used in 21 trials were sourced from the same pharmaceutical company, with a consistent batch number (See ***Background*** section). Furthermore, the content of each botanical drug in the ZRAS manufactured by this pharmaceutical company is consistent with the description of ZRAS in the Chinese Pharmacopoeia (https://ydz.chp.org.cn/#/main). The remaining trial was conducted in Australia, where the ZRAS used was manufactured by a local pharmaceutical company. However, the author of that trial highlighted that this company adhered to the standards of ZRAS described in the Chinese Pharmacopoeia during prescription production. Based on this consistency of sourcing and content, it can be considered that the prescriptions used in all trials are consistent and comparable.

All studies, except three (Liu and Nan, 2009; Zhong, 2018; Lu et al., 2023), reported AEs. Fatigue was the only AE associated with ZRAS with an incidence >3%, while eight types of AEs provoked by hypnotics had an incidence exceeding 3%. Although fatigue and dizziness were prevalent across all groups, the reporting rates were significantly lower in either ZRAS or ZRAS combined with hypnotic groups compared to the hypnotic group (6.2% in ZRAS *Vs*. 4.7% in combined group *vs*. 14.0% in hypnotic for fatigue; 2.8% in ZRAS *vs*. 2.9% in combined group *vs*. 11.2% in hypnotic for dizziness). The incidence of other symptoms such as dry mouth/bitter taste, excessive daytime drowsiness/sleepiness, gastrointestinal symptoms and headache/head painful distension was also lower in the combined group compared to the hypnotic group (Supplementary Appendix S5).

### 5.2 Study quality evaluation

#### 5.2.1 Assessing RCTs quality: ROB 2.0 indicates moderate to high ROB

**FIGURE 2:**
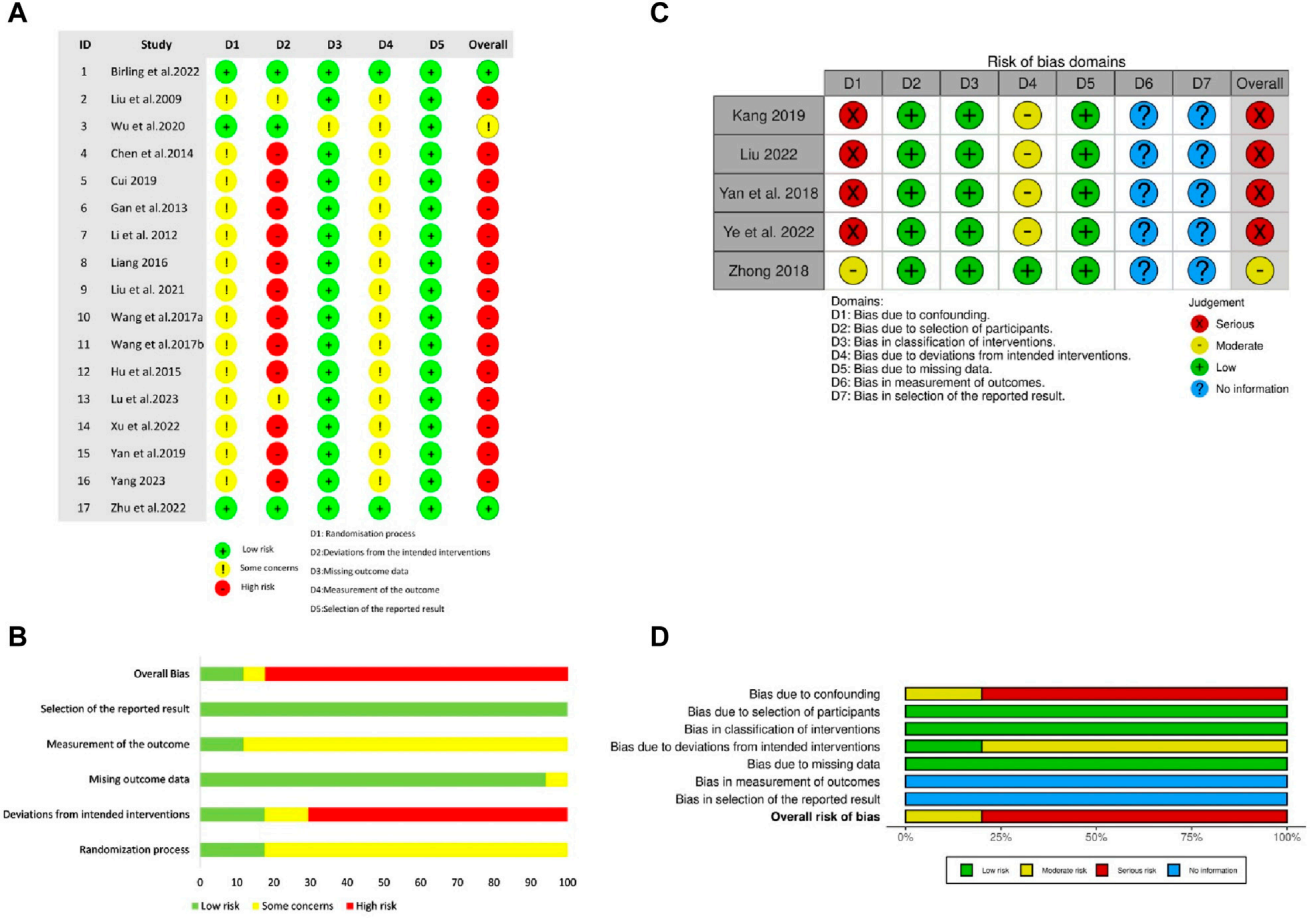
The risk of methodological bias in the included studies **(A)** Risk of Bias summary for RCTs **(B)** Risk of Bias graph for RCTs. The methodological quality of RCTs was appraised using Revised Cochrane Risk of Bias tool for randomised trials (ROB 2.0) **(C)** Risk of Bias graph for NRCTs **(D)** Risk of Bias summary for NRCTs. The methodological quality of NRCTs was appraised using Cochrane Risk of Bias in Non-randomized Studies-of Interventions (ROBINS-I).

Among the included RCTs, only three adequately described randomization methods, and employed valid allocation concealment using sealed blinding codes (Wu and Jiang, 2020; Birling et al., 2022; Zhu et al., 2022). They were also judged as having low RoB on the “deviations from intended interventions” domain due to participant-personnel double-blinding achieved via placebo-ZRAS capsules and/or placebo-hypnotics provided by pharmaceutical companies. Two studies were of some concern in this domain as only participants were blinded (Liu and Nan, 2009; Lu et al., 2023). Only two RCTs reported blinding of outcome evaluators (Birling et al., 2022; Zhu et al., 2022). One trial raised concerns in the “missing outcome data” domain due to an 8.3% dropout rate and exclusion of these cases from results analysis (Wu and Jiang, 2020). The remaining RCTs were appraised as having low RoB in this domain, either due to no participant withdrawal (Liu and Nan, 2009; Li and Gong, 2012; Gan et al., 2013; Chen et al., 2014; Hu and Sheng, 2015; Liang, 2016; Wang J. et al., 2017; Wang X. et al., 2017; Cui, 2019; Yan et al., 2019; Liu and Gong, 2021; Xu and Zhou, 2022; Lu et al., 2023; Yang, 2023) or by addressing missing data through both per protocol and intention-to-treat analyses (Birling et al., 2022; Zhu et al., 2022). All RCTs were rated as having low RoB in the “selection of reported outcomes” domain. Overall, two RCTs were rated as having low RoB, one had some concerns, and the rest were considered as having high RoB (Figures 2A, B).

#### 5.2.2 Assessing NRCTs quality: ROBINS-I indicates moderate to serious ROB

Regarding NRCTs, four (Yan et al., 2018; Kang, 2019; Liu, 2022; Ye and Lin, 2022) were assessed as having serious RoB in the “confounding” and moderate RoB in “deviations from intended interventions” domains because of potential impacts of confounding factors and intentional interventions on results. No bias existed in the participant selection. Given all participants completed the entire intervention without classification bias in this process, all included NRCTs were judged as low ROB in both “classification of interventions” and “missing data” domains. None of the NRCTs provided trial pre-registration information or described blinding of outcome assessors. Overall, one NRCT had moderate ROB, and the remaining were rated as having serious ROB (Figures 2C, D). 

### 5.3 Analyses of outcome measures

#### 5.3.1 ZRAS *vs* placebo-ZRAS: inconsistent findings detected

Three RCTs (*participants* = 385) addressed this comparison. Since none of the outcomes were utilized across all three studies, the results were only qualitatively described. Among these trials, two reported a significant reduction in PSQI scores with ZRAS (*p* < 0.05), highlighting its superiority over placebo-ZRAS (*p* < 0.05). However, another trial indicated a non-significant effect of ZRAS on ISI scores (*p* > 0.05), and found no statistical difference in ISI scores between ZRAS and placebo-ZRAS (*p* > 0.05). All three trials acknowledged the safety and tolerability of ZRAS without any significant adverse events.

#### 5.3.2 ZRAS *vs* hypnotic

Eleven trials (*participants* = 938) fell into this comparison and they were all RCTs. Meta-analyses were conducted for PSQI scores and clinical efficacy rate.

##### 5.3.2.1 PSQI global scores: no significant differences detected

**FIGURE 3:**
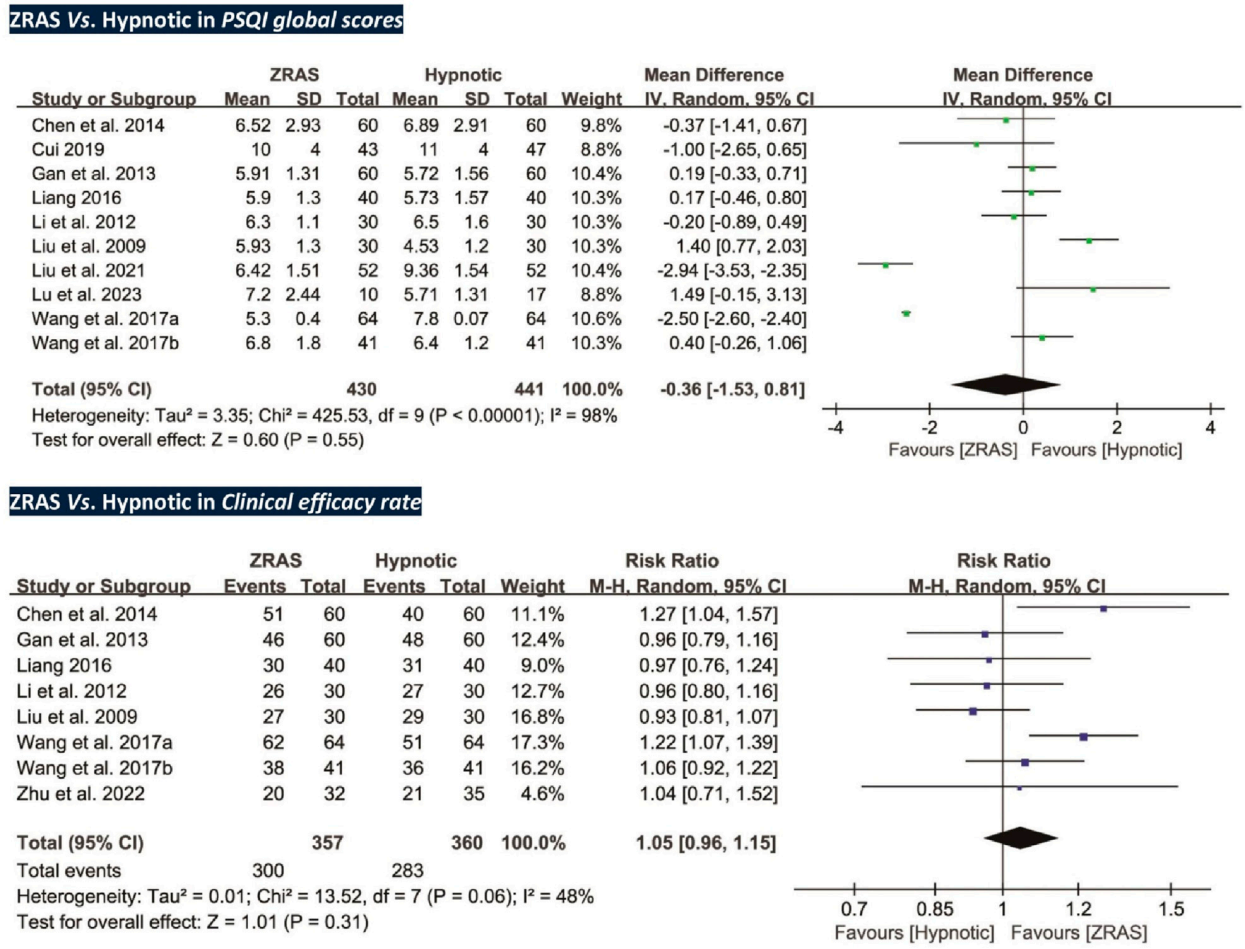
Forest plots of PSQI global scores and clinical efficacy rate (ZRAS *vs.* Hypnotic).

Ten trials (*participants* = 871) compared PSQI scores between ZRAS and hypnotic, revealing no significant differences [*MD* = −0.36, 95%*CI* (−1.53, 0.81), *p* = 0.55] (Figure 3). The *Z*-curve for PSQI in TSA exceeded the required information size (650), affirming the sufficiency of sample size in generating the current results (Supplementary Appendix S6).

Subgroup analyses were performed due to high heterogeneity. A significant interaction effect was identified between different hypnotics used in the controls (*Chi* ^*2*^ statistic 7.98, *df* = 1, *p* < 0.01). There was no significant difference between ZRAS and benzodiazepines in reducing PSQI scores [*MD* = 0.39, 95%*CI* (−0.12, 0.91), *p* = 0.13]. Whereas, ZRAS induced a more significant reduction in PSQI scores compared to Z-drugs [*MD* = −1.31, 95%*CI* (−2.37, −0.24), *p* = 0.02]. No interaction was noted in other subgroups (Supplementary Appendix S7).

Meta-regression indicated a potential and weak association of heterogeneity with study sample size (*I* ^*2*^ = 93.95%, *Tau* ^*2*^ = 1.29, *p* = 0.04), while publication year (*I* ^*2*^ = 96.15%, *Tau* ^*2*^ = 1.82, *p* = 0.26), therapeutic dosage (*I* ^*2*^ = 96.29%, *Tau* ^*2*^ = 1.80, *p* = 0.20), dosage forms of ZRAS (*I* ^*2*^ = 98.07%, *Tau* ^*2*^ = 2.30, *p* = 1.00), and hypnotics in the controls (*I* ^*2*^ = 86.08%, *Tau* ^*2*^ = 1.29, *p* = 0.07) could not explain heterogeneity (Supplementary Appendix S8).

Sensitivity analysis revealed minimal impact of individual trials on the pooled estimate effects of PSQI scores, suggesting overall robustness of the results (Supplementary Appendix S9).

##### 5.3.2.2 Clinical efficacy rate: no significant differences detected

Eight trials (*participants* = 717) employed clinical efficacy rate as an outcome (Liu and Nan, 2009; Li and Gong, 2012; Gan et al., 2013; Chen et al., 2014; Liang, 2016; Wang J. et al., 2017; Wang X. et al., 2017; Zhu et al., 2022). However, no significant differences were found between the groups [*RR* = 1.05, 95%*CI* (0.96, 1.15), *p* = 0.31] (Figure 3).

#### 5.3.3 ZRAS combined with hypnotic *vs* hypnotic

This category comprised 11 trials (*participants* = 884), involving RCTs and NRCTs. Meta-analysis was carried out for PSQI, clinical efficacy rate, and polysomnographically-recorded total sleep time, sleep onset latency and number of awakenings.

##### 5.3.3.1 PSQI global scores: ZRAS combined with hypnotic significantly reduces PSQI scores compared to hypnotic alone

**FIGURE 4:**
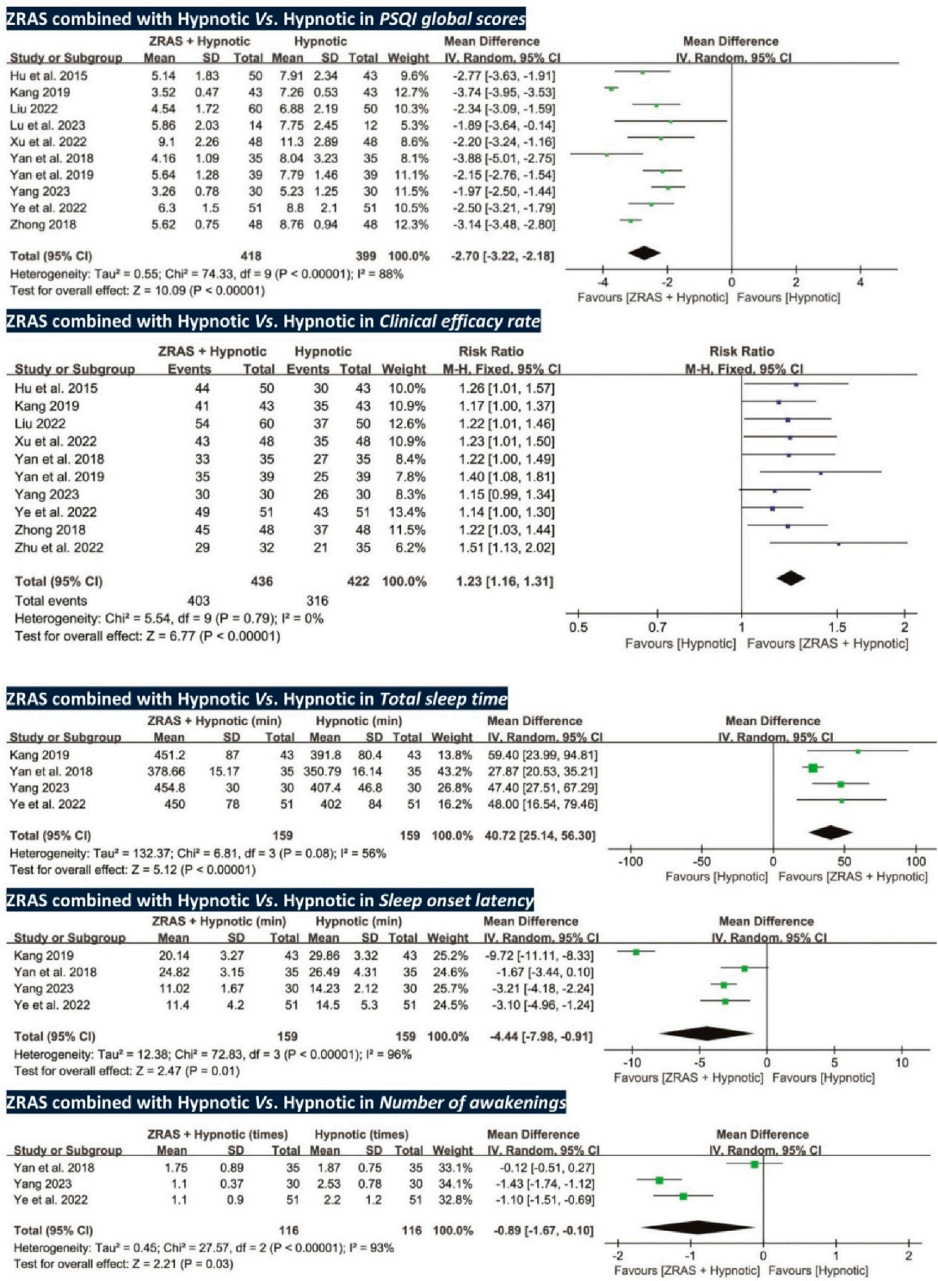
Forest plots of PSQI global scores, clinical efficacy rate, and polysomnography -recorded total sleep time, sleep onset latency and number of awakenings (ZRAS^+^ Hypnotic vs*.* Hypnotic).

All trials, except one (Zhu et al., 2022), used PSQI scores as an outcome (*participants* = 817). Pooled analysis favored ZRAS combined with hypnotic in reducing PSQI scores [*MD* = −2.70, 95%*CI* (−3.22, −2.18), *p* < 0.01] (Figure 4). The adequacy of sample size in this comparison was validated by TSA (Supplementary Appendix S10).

Subgroup analyses identified a significant interaction effect between different study types (*Chi* ^*2*^ statistic 8.30, *df* = 1, *p* < 0.01). In both RCTs and NRCTs, combined therapies showed more significant effects than hypnotic alone in reducing PSQI scores, with no heterogeneity in RCT design (*I* ^*2*^ = 0) (Supplementary Appendix S11). No interaction was identified in other subgroups.

Meta-regression indicated a potential and weak link of heterogeneity to study type (*I* ^*2*^ = 72.27%, *Tau* ^*2*^ = 0.19, *p* = 0.03), but not to publication year (*I* ^*2*^ = 84.06%, *Tau* ^*2*^ = 0.27, *p* = 0.09), study sample size (*I* ^*2*^ = 88.71%, *Tau* ^*2*^ = 0.40, *p* = 0.60), therapeutic dosage (*I* ^*2*^ = 87.14%, *Tau* ^*2*^ = 0.38, *p* = 0.42), dosage forms of ZRAS (*I* ^*2*^ = 81.21%, *Tau* ^*2*^ = 0.30, *p* = 0.19), and hypnotics in the controls (*I* ^*2*^ = 81.62%, *Tau* ^*2*^ = 0.41, *p* = 0.76) (Supplementary Appendix S12).

Sensitivity analysis confirmed the overall robustness of the results (Supplementary Appendix S13).

##### 5.3.3.2 Objective sleep parameters: ZRAS combined with hypnotic significantly improve sleep parameters compared to hypnotic alone

Total sleep time and sleep onset latency were assessed in four RCTs (*participants* = 318) (Yan et al., 2018; Kang, 2019; Ye and Lin, 2022; Yang, 2023); number of awakenings was assessed in three RCTs (*participants* = 232) (Yan et al., 2018; Ye and Lin, 2022; Yang, 2023). These parameters, collected via polysomnography, favored ZRAS combined with hypnotic in increasing total sleep time [*MD* = 40.72 *min*, 95%*CI* (25.14, 56.30), *p* < 0.01], shortening sleep onset latency [*MD* = −4.44 *min*, 95%*CI* (−7.98, −0.91), *p* = 0.01], and reducing number of awakenings [*MD* = −0.89 *times*, 95%*CI* (−1.67, −0.10), *p* = 0.03] (Figure 4). Subgroup analyses for total sleep time and sleep onset latency found no interactions.

##### 5.3.3.3 Clinical efficacy rate: ZRAS combined with hypnotic significantly improve clinical efficacy rate compared to hypnotic alone

Ten RCTs (*participants* = 858) used clinical efficacy rate as an outcome. The results favored ZRAS combined with hypnotic, showing a higher clinical efficacy rate than administering hypnotic alone [*RR* = 1.23, 95%*CI* (1.16, 1.31), *p* < 0.01] (Figure 4).

#### 5.3.4 ZRAS *vs* CBT-i, or ZRAS combined with CBTi *vs* CBTi: no trials addressed these comparisons

No trials were under these two comparisons.

### 5.4 Publication bias test

We performed publication bias test based on PSQI in both comparisons, revealing statistically significant effect (*p* < 0.01 for ZRAS *Vs*. hypnotic; and *p* = 0.02 for ZRAS combined with hypnotic *Vs*. hypnotic) (Supplementary Appendix S14).

### 5.5 Certainty and quality of evidence

The certainty and quality of evidence from meta-analyses of seven outcomes are outlined in Supplementary Appendix S15. In pursuance of the GRADE system, the quality of evidence ranged between very low and low ratings, with six outcomes rated as “Very low” and one rated as “Low.” The predominant factor contributing to degradation was the risk of bias within the trials included. Furthermore, some of the included trials were NRCT rather than RCT designs, contributing to initial degradation.

## 6 Discussion

### 6.1 Summary of findings

Our review reflected the current knowledge state regarding using ZRAS for PI. ZRAS was comparable to benzodiazepines but superior to Z-drugs in reducing PSQI global scores. Compared to hypnotics alone, the addition of ZRAS further reduced PSQI scores by 2.2–3.2 points, which was clinically significant (Zhao et al., 2022). Such addition of ZRAS also further extended total sleep time, shortened sleep onset latency and reduced number of awakenings. The cumulative sample size for meta-synthesis was sufficient. Nevertheless, the evidence supporting these positive results had very-low-to-low quality due to insufficient blinding and underuse of RCT design. Comparative efficacy between ZRAS and CBT-i, or combined ZRAS and CBT-i versus CBT-i alone, could not be determined due to lack of available data. ZRAS demonstrated good tolerability with AEs markedly lower than those associated with hypnotics; all ZRAS-related AEs were under 3%, except for fatigue, which reached 6.2%. Overall, ZRAS is safe for management of PI, while its efficacy cannot be definitely concluded due to quality shortfalls in most of the included trials.

### 6.2 Strengths, limitations, and comparison with previous systematic reviews

We noticed four Chinese (Ji et al., 2019; Yuan, 2019; Wang et al., 2020; Zhu and Wang, 2020) and two English (Birling et al., 2020; Chen et al., 2020) available systematic reviews and/or meta-analyses with similar theme. Five of them only reviewed ZRAS capsules, omitting other dosage forms (Ji et al., 2019; Yuan, 2019; Chen et al., 2020; Wang et al., 2020; Zhu and Wang, 2020). In these reviews, inappropriate inclusion of trials on compounded ZRAS capsule, those lacking valid diagnostic criteria, and/or those without internationally recognized sleep scales/questionnaires for quantitative synthesis also introduced extra variability, making it difficult to interpret the results. The remaining one review even included studies targeting insomnia secondary to schizophrenia and hypertension (Birling et al., 2020), further complicating interpretation. All six reviews were conducted at least 5 years ago, whereas our study incorporated the latest evidence from recent years.

Our review, with updated retrieval and stricter selection criteria, included more trials and reduced variability. Other strengths included: 1) we provided evidence that ZRAS enhanced objective sleep parameters, a critical clinical issue inadequately addressed in previous reviews; 2) we appraised the methodology in different types of trial using the eligible tools, an aspect ignored in two earlier reviews (Ji et al., 2019; Birling et al., 2020); and 3) the GRADE framework, employed in our review, was not previously used in any ZRAS-related reviews to assess evidence quality.

However, limitations exist. First, the poor methodological quality of included trials seriously undermines evidence reliability. Second, high heterogeneity could not be fully explained despite use of subgroup, meta-regression and sensitivity analyses. This, however, does not imply that low-quality trials should be excluded. A previous methodological study highlighted that there was a danger with any approach to excluding trials from systematic review because there was no clear-cut distinction between high- and low-quality trials, and reviewers can only know that they are susceptible to bias but can never know for sure whether a trial is biased (Harvey and Dijkers, 2019). Furthermore, TSA analysis demonstrated that the sample size for meta-synthesis was sufficient; and subgroup, meta-regression, and sensitivity analyses confirmed the robustness of the results, i.e., none of the potential influencing factors altered the overall findings. Finally, 21/22 (95.5%) trials included were conducted in China, possibly inflating effectiveness of TCM therapies due to cultural confidence among patients (Zhao et al., 2022). Chinese researchers might also be more inclined to report positive findings. This is evidenced by the significant publication bias we identified (Supplementary Appendix S14). The applicability of present findings beyond Chinese communities is limited. ZRAS has been listed with the Australian Registry of Therapeutic Goods under the name “Zao Ren An Shen” (AUST L301484) (Birling et al., 2022), providing a foundation for studying its applicability to diverse consumer populations.

### 6.3 Interpretation of findings

CBT-i is the front-line treatment for PI (Zhao et al., 2021), yet its limited accessibility makes CAM remedies more appealing in some regions where traditional medicine practices are widely accepted (Ell et al., 2023). Chinese medicines have been used to manage insomnia for over 2,000 years (Ni et al., 2015). A National Health Interview Survey estimates that over 1.6 million adults in United States use CAM to manage insomnia or trouble sleeping (Pearson et al., 2006). Furthermore, the use of botanical drugs was found to be the most prevalent and popular, with 49.2% users reporting significant improvement (Pearson et al., 2006). No studies available compared ZRAS with CBT-i, warranting further investigation into their comparative efficacy. Benzodiazepines and Z-drugs, most commonly prescribed for insomnia, raise concerns regarding dependency and safety (Leach and Page, 2015). Benzodiazepines may cause dizziness, headaches, lethargy, nightmares, daytime fatigue, ataxia, nausea, and/or falls; and Z-drugs are associated with dizziness, somnolence, falls, headaches and migraine, nausea and emesis, and/or diarrhea (Leach and Page, 2015; Madari et al., 2021). Some of these AEs were confirmed in our review (Supplementary Appendix S5). CCPP, as a CAM product, also has some adverse drug reactions (Chan et al., 2015). However, the reported AEs caused by even combined ZRAS and hypnotics were fewer than those of hypnotics, implying a clinically valuable option of adding ZRAS to benzodiazepines/Z-drugs to optimize effectiveness while minimizing AEs. Such co-administration of conventional and Chinese medicines has already been established as a routine modality in modern China for managing sleep disturbances (Ni et al., 2015). While this model remains less prevalent in Western nations, our findings provide valuable insights from China’s experience for policymakers of these countries to seriously consider the WHO’s recommendation to integrate traditional medicine into the national healthcare system (von Schoen-Angerer et al., 2023). Moreover, standardization in preparation endows CCPP with advantages such as stable quality, heightened safety, rapid absorption, and enhanced bioavailability (Zhang et al., 2020). Compared to Chinese medicinal decoctions, CCPP not only offer greater convenience for patients in terms of administration, transportation, and storage (Zhang et al., 2020), but they also facilitate prescription issuance for physicians (Chen et al., 2012). These advantages render CCPP more suitable for promotion in Western countries, including the United States and European Union member states (Chen et al., 2012).

By using the sleep-deprived animal models, the possible mechanisms of ZRAS in improving PI have been investigated. Studies have found that ZRAS reduced the sleep onset latency (Chen and Shan, 2020) and wake after sleep onset (Wang et al., 2023), while prolonged the total sleep time (Wang et al., 2023) of sleep-deprived rodents. This improvement in sleep quality was associated with reduced dopamine (Li, 2020), increased serotonin (Li, 2020), and/or increased adenosine A1/A2 receptor levels (Chen and Shan, 2020) in the brain of the rodents. Additionally, network pharmacology analyses demonstrated that ZRAS improved sleep through multiple targets and pathways, including the modulation of neurotransmitters (Yang et al., 2020; Zhu, 2021), protein phosphorylation (Zhu, 2021), and tryptophan metabolism (Yang et al., 2020).

Studies have also focused on the individual metabolites that ZARS contains. For example, jujubosides, jujuboside A, and jujuboside B, which are saponins from Ziziphi spinosae semen, have been reported to have sedative and hypnotic effects (Bian et al., 2021). The hypnotic effect of jujubosides is believed to be mediated through the serotonergic system (Cao et al., 2010). Extracts from Salviae miltiorrhizae radix et rhizoma have also been documented to exert sedation effect (Lobina et al., 2018). Tanshinones, the principal bioactive metabolites of Salviae miltiorrhizae radix et rhizoma (Yang et al., 2019), demonstrate significant sedative-hypnotic effects (Fang et al., 2010). In addition, animal studies demonstrated that schizandrin, a major metabolite of Schisandrae chinensis fructus, could pass through the blood-brain barrier and exhibited sedative and hypnotic bioactivity, potentially mediated by its modulation of the serotonergic system (Zhang et al., 2018). Zheng *et al.* had investigated the synergistic mechanism of multiple metabolites in the combination of Ziziphi spinosae semen and Schisandrae chinensis fructus for insomnia treatment. Utilizing network pharmacology-based and molecular docking approach, the research group identified 41 target-disease related genes of this combination and found that neuroactive ligand-receptor interactions is a key mechanism underlying the efficacy of this drug combination in treating insomnia (Zheng et al., 2022). Similarly, another animal study revealed a synergistic effect of combining Ziziphi spinosae semen and Salviae miltiorrhizae radix et rhizoma. This would enhance sleep duration and reduce sleep onset latency in rodent models (Fang et al., 2010).

Only three reviewed trials included follow-ups (two- or 4-week) (Birling et al., 2022; Zhu et al., 2022), hindering the determination of the medium-to-long-term efficacy of ZRAS. Investigating issues commonly occur in hypnotics—such as dependence, withdrawal symptoms, and rebound insomnia (Voshaar et al., 2004; Schifano et al., 2019)—when using ZRAS is essential. While few trials included suggested that ZRAS alleviated concomitant symptoms of PI, such as fatigue (Liu and Gong, 2021), anxiety (Kang, 2019; Liu and Gong, 2021) and depression (Kang, 2019), inadequate data impedes quantitative analysis. Studying ZRAS’s potential to address these accompanying symptoms is crucial, particularly for CAM users with motivation for more holistic care by addressing multivariate symptoms (Satija and Bhatnagar, 2017; Kemppainen et al., 2018). This appears to be promising because TCM tends to concentrate on the overall functional wellbeing rather than the disease defined by specific pathological changes only (Jiang, 2005). Previous clinical (Yu et al., 2022) and pre-clinical (Wang et al., 2021) studies also showed that ZRAS exerted both anxiolytic and sleep-promoting effects in patients/animal models with comorbid anxiety and insomnia.

The Australian study previously cited was the sole trial suggesting that ZRAS might not effectively improve PI (Birling et al., 2022). The investigators claimed that TCM syndrome pattern did not influence treatment outcome, as there was no statistical difference in TCM pattern scores between ZRAS and placebo-ZRAS after treatment (Birling et al., 2022). However, we hold a different perspective. Syndrome differentiation-based treatment is a fundamental principle guiding Chinese medicine prescriptions (Jiang et al., 2012), and is believed to provide better efficacy (Yeung et al., 2012). The PPRC (https://ydz.chp.org.cn/#/main) states that ZRAS is best suited for insomnia with “deficiency of *Heart*-blood” pattern. In the Australian study: 1) not all patients met this pattern; 2) the pattern was diagnosed by a single investigator and quantified using a self-reported questionnaire without psychometric properties; 3) the statistical power was insufficient to infer the pattern’s impact on efficacy, considering only 38 patients with multiple patterns in the experimental group (Birling et al., 2019; Birling et al., 2022). Evidently, factors influencing the trial design for Chinese medicine may not solely stem from differences between Eastern and Western cultures, but also from disparities among scholars’ fundamental understandings of TCM, particularly regarding the awareness of syndrome differentiation. Modern western medicine only emphasizes the necessity of clear diagnosis for treatment, while the application of Chinese medicine requires not only a clear diagnosis of the disease but also of the associated TCM syndromes. Even if a patient is diagnosed with PI, for instance, different TCM syndromes may necessitate distinct treatments. For instance, The *Pharmacopoeia* (https://ydz.chp.org.cn/#/main) explicitly states that for PI (deficiency of *Heart*-blood), ZRAS are recommended, whereas for PI patients diagnosed as “deficiency of *Kidney* essence” and “stagnation of *Qi* due to depression of the *Live*r,” An Shen Bu Nao solution and Jie Yu An Shen granules are respectively recommended. In addition to randomization, double-blinding and placebo-control design, future studies should also focus on participants with fixed pattern (deficiency of *Heart*-blood) and increase sample size to minimize statistical error.

Although ZRAS may be a promising alternative option for PI, the current favorable findings should be approached cautiously due to certain concerns about their reliability. First, most of the trials (*n* = 19, 86.4%) did not report any attrition (Figure 2), which is unusual in insomnia-related clinical trials (Birling et al., 2020). Secondly, only two included trials implemented adequate blinding. Adequate blinding in RCTs for participants, personnel and assessors is essential to minimize risk of bias, especially when primary outcomes relied heavily on subjective assessments by participants or clinicians. Even objective outcomes such as polysomnography-measured parameters can be compromised as it is unclear whether the technicians who interpreted the measurements were aware or not of the allocation (Birling et al., 2020). Unlike acupuncture trials, achieving proper blinding with placebos in drug trials is feasible. Whereas, there are also emerging perspective advocating for unblinded pragmatic trials due to their emphasis on practical applicability and generalizability, which can improve the external validity of real-world trials beyond treatment effects, although this approach is a major contributor to the risk of bias (Sox and Lewis, 2016). To reconcile these differing views, we recommend conducting blinded RCTs as well as unblinded real-world studies separately. Combining results from both research paradigms may lead to a more reliable and impartial assessment of ZRAS. Finally, the dearth of pre-registration information compromised the transparency of the trial results. This is also a primary factor contributing to publication bias risk, as it leaves open the possibility of selective reporting by authors. Therefore, pre-registration and protocol uploading in future studies are necessary.

## 7 Conclusion

The studies reviewed in this analysis demonstrate a preference for ZRAS over benzodiazepines and Z-drugs in terms of short-term efficacy. Moreover, combining ZRAS with benzodiazepines/Z-drugs has also yield greater therapeutic effect with fewer AEs. However, the reliability of these findings is significantly compromised by the methodological drawbacks of the included studies. Furthermore, the limited number of trials providing follow-up data and/or assessing accompanying symptoms of PI, such as fatigue, depression, anxiety, etc., precludes quantitative analysis. Therefore, the medium-to-long-term efficacy of ZRAS in treating PI and associated symptoms remains uncertain. Given the potential benefits of ZRAS, well-designed RCTs with extended follow-up periods and comprehensive outcomes are warranted. Nevertheless, the current body of evidence does not offer sufficient support to conclusively endorse the use of ZRAS for PI treatment.

## Data Availability

The original contributions presented in the study are included in the article/Supplementary Material, further inquiries can be directed to the corresponding authors.

## Abbreviations

AE(s), adverse event(s); AIS, Athens Insomnia Scale; CAM, Complementary and alternative medicine; CBT-i, Cognitive behavioral therapy for insomnia; CCPP, Commercial Chinese polyherbal preparation; CENTRAL, Cochrane Central Register of Controlled Trials; CFDA, China Food and Drug Administration; CQVIP, Chongqing VIP database; GRADE, Grades of Recommendation, Assessment, Development, and Evaluation; ISI, Insomnia Severity Index; NRCT(s), Non-randomized controlled clinical trial(s); PI, Primary insomnia; PPRC, Pharmacopoeia of the People’s Republic of China; PRISMA, Preferred Reporting Items for Systematic Reviews and Meta-Analyses; PSQI, Pittsburgh Sleep Quality Index; RCT(s), Randomized control trial(s); ROB, Risk of Bias; ROB 2.0, Revised Cochrane Risk of Bias tool for randomised trials; ROBINS-I, Cochrane Risk of Bias in Non-randomized Studies-of Interventions; SCMM, Shennong’s Classic of Materia Medica; SinoMed, China biomedical literature service system; TCM, Traditional Chinese medicine; TSA, Trial sequential analysis; WHO, World Health Organization; ZRAS, Zao Ren An Shen.
