# Supplementary material for: Commercial Chinese polyherbal preparation Zao Ren An Shen prescription for primary insomnia: a systematic review with meta-analysis and trial sequential analysis
Source: Front Pharmacol. 2024 Jun 18;15:1376637. doi: 10.3389/fphar.2024.1376637 (PMC11218554; doi:10.3389/fphar.2024.1376637)
Supplement: Supplementary file 1 [file DataSheet1.pdf]

Supplementary Materials

Commercial Chinese polyherbal preparation Zao Ren An Shen prescription for primary insomnia: A systematic review with meta-analysis and trial sequential analysis

Fei-Yi Zhao[a,b†], Peijie Xu[c†], Gerard A. Kennedy[d,e,f†], Zhen Zheng[e], Wen-Jing Zhang[b], Jia-Yi Zhu[g], Yuen-Shan Ho[h*], Li-Ping Yue[a*], Qiang-Qiang Fu[g*], Russell Conduit[e]

[a] *Department of Nursing, School of International Medical Technology, Shanghai Sanda University, Shanghai 201209, China*
[b] *Shanghai Municipal Hospital of Traditional Chinese Medicine, Shanghai University of Traditional Chinese Medicine, Shanghai 200071, China*
[c] *School of Computing Technologies, RMIT University, Melbourne, VIC, 3000, Australia*
[d] *Institute of Health and Wellbeing, Federation University, Mount Helen, Victoria, Australia*
[e] *School of Health and Biomedical Sciences, RMIT University, Bundoora Victoria, 3083, Australia*
[f] *Institute for Breathing and Sleep, Austin Health, Heidelberg, Victoria, 3084, Australia*
[g] *Yangpu Hospital, School of Medicine, Tongji University, Shanghai, China*
[h] *School of Nursing, Faculty of Health and Social Sciences, The Hong Kong Polytechnic University, Hong Kong SAR, China*

†These authors contributed equally to this manuscript and should be considered co-first authors.

Correspondence should be addressed to Yuen-Shan Ho, janice.ys.ho@polyu.edu.hk; Li-Ping Yue, lpyue@sandau.edu.cn; and Qiang-Qiang Fu, qiangqiang.fu@tongji.edu.cn

*Correspondence address:
Associate Professor, Yuen-Shan Ho, School of Nursing, School of Nursing, Faculty of Health and Social Sciences, The Hong Kong Polytechnic University, Hong Kong SAR, CHINA Tel: (+852) 2766-6410; Fax: (+852) 2364-9663; E-mail: janice.ys.ho@polyu.edu.hk

Associate Professor, Li-Ping Yue, Department of Nursing, School of International Medical Technology, Shanghai Sanda University, Shanghai 201209, CHINA Tel: (+ 86) 021-50210894; Fax: (+ 86) 021-50210895; Email: lpyue@sandau.edu.cn

Associate Professor, Qiang-Qiang Fu, Yangpu Hospital, School of Medicine, Tongji University, Shanghai 200090, CHINA Tel: (+ 86) 021-6569 0520; Fax: (+ 86) 021-6569 6249; Email: qiangqiang.fu@tongji.edu.cn

Appendix 1 Detailed description of ZRAS capsule and ZRAS granules in *Pharmacopoeia of the People's Republic of China*, 2020 version

	ZRAS capsule	ZRAS granules
Component	*Ziziphus jujuba* Mill. var. *spinosa* (Bunge) Hu ex H. F. Chou [Rhamnaceae; *Ziziphi spinosae semen*]<br>*Schisandra chinensis* (Turcz.) Baill. [Schisandraceae;]<br>*Salvia miltiorrhiza* Bunge [Lamiaceae; *Salviae miltiorrhizae radix et rhizoma*]	*Ziziphus jujuba* Mill. var. *spinosa* (Bunge) Hu ex H. F. Chou [Rhamnaceae; *Ziziphi spinosae semen*]<br>*Schisandra chinensis* (Turcz.) Baill. [Schisandraceae; *Schisandrae chinensis fructus*]<br>*Salvia miltiorrhiza* Bunge [Lamiaceae; *Salviae miltiorrhizae radix et rhizoma*]
Prescription	*Ziziphi spinosae semen* 1425g<br>*Schisandrae chinensis fructus* 285g<br>*Salviae miltiorrhizae radix et rhizoma* 285g	*Ziziphi spinosae semen* 1425g<br>*Schisandrae chinensis fructus* 285g<br>*Salviae miltiorrhizae radix et rhizoma* 285g
Preparation (formulation processes)	The medicinal formula involves reflux extraction of three botanical drugs, namely *Ziziphi spinosae semen*, *Schisandrae chinensis fructus*, and *Salviae miltiorrhizae radix et rhizoma*, in 75% ethanol for 2 hours. The filtrate is then collected and set aside. The residue is subjected to a second reflux with 60% ethanol for 1 hour, filtered, and combined with the previous filtrate. The ethanol is reclaimed from the combined filtrate and concentrated to a thick paste with a relative density of 1.30 (60°C). The residue undergoes further decoction with water, lasting 2 hours for the first time and 1 hour for the second. The resulting filtrates are combined, concentrated to a thick paste with a relative density of 1.30 (60°C), added to the previous paste, further concentrated to a relative density of 1.40 (60°C), and then mixed with an appropriate amount of starch. The mixture is formed into granules, dried, encapsulated, resulting in 1000 capsules.	The medicinal formula involves the decoction of three botanical drugs, namely *Ziziphi spinosae semen*, *Schisandrae chinensis fructus*, and *Salviae miltiorrhizae radix et rhizoma*, in water for two times, each lasting 2 hours. The decoction liquids are combined, filtered, and the filtrate is concentrated to a clear paste with a relative density ranging from 1.05 to 1.20. An appropriate amount of pregelatinized starch is added, mixed thoroughly, and the resulting mixture is formed into granules, dried, yielding a total of 1000g.
Characteristics	The product is presented in the form of hard capsules containing granules and powder ranging in color from yellowish-brown to brown. It possesses a fragrant odor, with a sour and slightly bitter taste.	The product is presented in the form of granules ranging in color from yellowish-brown to brown. It possesses a fragrant odor, with a sour and slightly bitter taste.
Therapeutic functions	Nourishing Blood and calming the mind. Indicated for insomnia, forgetfulness, restlessness, and dizziness caused by deficient *Blood* in the *Heart*; also applicable for neuroasthenic symptoms.	Nourishing Blood and calming the mind. Indicated for insomnia, forgetfulness, restlessness, and dizziness caused by deficient *Blood* in the *Heart*; also applicable for neuroasthenic symptoms.
Dosage and administration	Oral administration. Take 5 capsules at a time, once daily, before bedtime.	To be taken by dissolving in hot water. Consume 1 sachet at a time, once daily, before bedtime.
Precautions	Caution should be exercised in pregnant women.	Caution should be exercised in pregnant women.

Specifications	Each capsule contains 0.45g.	Each sachet contains 5g.
Storage	Seal tightly.	Seal tightly.

Appendix 2 Additional notes on *Method* section

2.1 Standard diagnostic criteria

- Diagnostic and Statistical Manual of Mental Disorders, Fourth Edition (DSM-IV)
- Diagnostic and Statistical Manual of Mental Disorders, Fifth Edition (DSM-V)
- International Classification of Diseases, 10th Edition (ICD-10)
- International Classification of Diseases and Related Health Problems, 11th Edition (ICD-11)
- International Classification of Sleep Disorders, Third Edition (ICSD-3)
- Chinese Classification of Mental Disorders, Second Edition (CCMD-2)
- Chinese Classification of Mental Disorders, Second Edition, Revised (CCMD-2-R)
- Chinese Classification of Mental Disorders, Third Edition (CCMD-3)
- Guideline for the Diagnosis and Treatment of Insomnia in Chinese Adults (GDTICA)

2.2 Search strategy for each database

Databases	Search strategy
MEDLINE (via PubMed)	#1 Zao Ren An Shen[Title/Abstract]<br>#2 Zaoren Anshen[Title/Abstract]<br>#3 ZRAS[Title/Abstract]<br>#4 #1 or #2 or #3<br>#5 insomnia[MeSH Terms]<br>#6 insomnia[Title/Abstract]<br>#7 "sleep Initiation and maintenance disorders"[Title/Abstract]<br>#8 sleep disorder[Title/Abstract]<br>#9 dyssomnias[Title/Abstract]<br>#10 sleep wake disorders[Title/Abstract]<br>#11 #5 or #6 or #7 or #8 or #9 or #10<br>#12 #4 and #11
Cochrane Central Register of Controlled Trials (CENTRAL)	#1 (Zao Ren An Shen):ti,ab,kw<br>#2 (Zaoren Anshen):ti,ab,kw<br>#3 (ZRAS):ti,ab,kw<br>#4 #1 or #2 or #3<br>#5 MeSH descriptor: [Sleep Initiation and Maintenance Disorders] explode all trees<br>#6 (insomnia):ti,ab,kw<br>#7 ("sleep Initiation and maintenance disorders"):ti,ab,kw<br>#8 (sleep disorder):ti,ab,kw<br>#9 (dyssomnias):ti,ab,kw<br>#10 (sleep wake disorders):ti,ab,kw<br>#11 #5 or #6 or #7 or #8 or #9 or #10<br>#12 #4 and #11
EMBASE	#1 'zao ren an shen':ti,ab,kw<br>#2 'zaoren anshen':ti,ab,kw<br>#3 'zras':ti,ab,kw<br>#4 #1 OR #2 OR #3<br>#5 'insomnia'/exp OR 'insomnia'

	#6 'insomnia':ti,ab,kw<br>#7 'sleep disorder':ti,ab,kw<br>#8 'dyssomnias':ti,ab,kw<br>#9 #5 OR #6 OR #7 OR #8<br>#10 #4 AND #9
China National Knowledge Infrastructure (CNKI)	(篇关摘：枣仁安神(模糊)) AND (篇关摘：失眠(模糊)) OR (篇关摘：不寐(模糊)) OR (篇关摘：原发性失眠(模糊)) OR (篇关摘：睡眠障碍(模糊)) AND (篇关摘：临床试验(模糊)) OR (篇关摘：随机对照试验(模糊)) OR (篇关摘：试验(模糊))
Wanfang database	题名或关键词:(枣仁安神) and 题名或关键词:(失眠) or 题名或关键词:(原发性失眠) or 题名或关键词:(不寐) or 题名或关键词:(睡眠障碍) and 题名或关键词:(临床) and 题名或关键词:(随机对照试验) and 题名或关键词:(试验)
Chongqing VIP database (CQVIP)	题名或关键词=枣仁安神 AND 题名或关键词=失眠+ 不寐 + 睡眠障碍 AND 题名或关键词=随机对照试验 + 随机对照实验 + 随机对照研究
China biomedical literature service system (SinoMed)	"枣仁安神"[中文标题:智能] AND "失眠"[核心字段:智能] OR "原发性失眠"[核心字段:智能] OR "睡眠障碍"[核心字段:智能] OR "不寐"[常用字段:智能] AND "临床试验"[常用字段:智能] OR "随机对照试验"[常用字段:智能] OR "试验"[常用字段:智能]
US ClinicalTrials.gov	(Zao Ren An Shen OR Zaoren Anshen) AND (insomnia sleep OR Initiation and maintenance disorders OR sleep disorder OR sleep wake disorders OR dyssomnias OR sleep disorder) AND (randomized clinical trials OR randomized controlled clinical trial OR randomized controlled trials OR randomized experiment OR rct)
WHO International clinical trials registry platform search portal	(Zao Ren An Shen OR Zaoren Anshen) AND (insomnia sleep OR Initiation and maintenance disorders OR sleep disorder OR sleep wake disorders OR dyssomnias OR sleep disorder) AND (randomized clinical trials OR randomized controlled clinical trial OR randomized controlled trials OR randomized experiment OR rct)

Appendix 3 Excluded studies with specific reasons

References	Reasons for exclusion
Feng JW. Effectiveness and safety of Zao Ren An Shen capsule in the treatment of insomnia [article in Chinese]. *Journal of North Pharmacy*, 2021; 18(5):129-130.	without internationally recognized, validated scales/questionnaires for quantifying insomnia
Liu HY, Chen FZ. Efficacy of Zao Ren An Shen capsule in the treatment of senile insomnia [article in Chinese]. *World Latest Medicine Information*, 2017; 17(2):71.	without clear diagnostic criteria for primary insomnia
Liu R. Clinical study of compound Zao Ren An Shen capsule combined with Zopiclone in the treatment of insomnia with *Heart-Kidney* imbalance syndrome and its effect on serum 5-HT, IL-1β and PGD2 [article in Chinese]. *Master thesis*, 2019; Anhui University of Chinese Medicine.	mismatch of ingredients in proprietary Chinese medicine
Ma YD, Li RH, Tao YJ, Sun LR, Hu GZ, Liu YF. Therapeutic effect of compound Zao Ren An Shen capsule in the treatment of insomnia and pharmacological research [article in Chinese]. *Journal of Integrated Traditional Chinese and Western Medicine*, 1989; (2):85-87, 68-69.	without internationally recognized, validated scales/questionnaires for quantifying insomnia
Pan ZY. Clinical study of Zao Ren An Shen capsule combined with Estazolam in the treatment of sleep disorders [article in Chinese]. *World Journal of Sleep Medicine*, 2021; 8(12):2098-2100.	depression-related insomnia
Qin GX, Jin HL, Lu GF. A controlled study of Zao Ren An Shen capsule in the treatment of insomnia [article in Chinese]. *Zhejiang Journal of Integrated Traditional Chinese and Western Medicine*, 2007(12):746-747.	without internationally recognized, validated scales/questionnaires for quantifying insomnia
Qin GX, Tian GQ, Gan JG. A controlled study of polysomnography in patients with insomnia. *Chinese Rural Health Service Administration*, 2015; 35(6):796-798.	without subjective sleep outcomes (any validated scale/questionnaire)
Ren YJ, Ni F. Efficacy of Zaoren Anshen capsule in the treatment of senile insomnia [article in Chinese]. *Journal of Guizhou University of Traditional Chinese Medicine*, 2007; (5):23-24.	without internationally recognized, validated scales/questionnaires for quantifying insomnia
Shao MX, Bao XY, Yao Y, Yan DJ, Wu SF, Zhu XY. 124 cases of senior insomnia patients treated with Zao Ren An Shen capsule [article in Chinese]. *Chinese Journal of Information on Traditional Chinese Medicine*, 1999; (3):33.	without clear diagnostic criteria for primary insomnia
Sun HH. Effectiveness of Zao Ren An Shen capsule in the treatment of insomnia [article in Chinese]. *World Latest Medicine Information*, 2019; 19(44):130.	without clear diagnostic criteria for primary insomnia
Wang SM, Liu JQ, Ma GF, Lan HY, Zhao ZR. Clinical study of Zao Ren An Shen tablet in the treatment of insomnia [article in Chinese]. *Hebei Journal of Traditional Chinese Medicine*, 2013; 35(8):1217-1219.	mismatch of ingredients in proprietary Chinese medicine
Wang Y, Hu DM. Comparative study of Zao Ren An Shen capsule and Diazepam in the treatment of insomnia [article in Chinese]. *Shanghai Journal of Traditional Chinese Medicine*, 1997; (12):38-39.	without clear diagnostic criteria for primary insomnia
Wu YF, Lv BJ, Teng JB. Clinical study of Zao Ren An Shen capsule combined with Estazolam tablets in the treatment of sleep disorders [article in	Use of non-standard diagnostic guideline for

Chinese]. *New Chinese Medicine*, 2019; 51(8):98-100.	insomnia
Xu C. A controlled study of Zao Ren An Shen and Alprazolam in the treatment of insomnia [article in Chinese]. *Inner Mongolia Journal of Traditional Chinese*, 2011; 30(23):3.	without internationally recognized, validated scales/questionnaires for quantifying insomnia
Xu C. A controlled study of Zao Ren An Shen and Zopiclone in the treatment of insomnia [article in Chinese]. *World Health Digest*, 2012; 9(6):46-47.	without internationally recognized, validated scales/questionnaires for quantifying insomnia
Zhang J. Clinical effect of Zao Ren An Shen capsule and Eszopiclone in the treatment of insomnia [article in Chinese]. *Medical Equipment*, 2016; 29(13):113.	without internationally recognized, validated scales/questionnaires for quantifying insomnia
Zhang SN, Zhang B, Li YJ, Chen YH. Effects of compound Zao Ren An Shen capsule on serum 5-HT, IL-1β and PGD2 among patients with primary insomnia [article in Chinese]. *China Health Care & Nutrition*, 2018; 28(34):75-76.	mismatch of ingredients in proprietary Chinese medicine
Zhang WT, Shi ZG, Sun Y. Efficacy of Zao Ren An Shen Capsule in the treatment of 32 cases of insomnia with deficiency of both *Heart* and *Spleen* syndrome [article in Chinese]. *China Pharmaceuticals*, 2007; (19):58.	without internationally recognized, validated scales/questionnaires for quantifying insomnia
Zhang YW. Effect of Zao Ren An Shen capsule in the treatment of insomnia [article in Chinese]. *Contemporary Medical Symposium*, 2019; 17(16):167-168.	without internationally recognized, validated scales/questionnaires for quantifying insomnia
Zhao B. Efficacy of Zao Ren An Shen liquid combined with Zopiclone in the treatment of insomnia [article in Chinese]. *China Health Care & Nutrition*, 2015; 25(13):355.	without internationally recognized, validated scales/questionnaires for quantifying insomnia
Zhao WM. Clinical study of compound Zao Ren An Shen capsule in the treatment of insomnia with *Heart-Liver* blood deficiency syndrome [article in Chinese]. *Master thesis*, 2017; Anhui University of Chinese Medicine.	mismatch of ingredients in proprietary Chinese medicine

Appendix 4 Grading criteria of clinical efficacy rate used in the included studies

Criteria	Details of guidelines	Involved studies	Frequency, *n* (%)
**Method 1**	***Healed***: sleep back to normal, TST > 6h	Liang, 2016; Yan et al., 2019; Liu, 2022; Xu and Zhou, 2022; Ye and Lin, 2022; Yang, 2023	6 (33.3)
	Significant efficacious: sleep improves, TST↑≥ 3h		
	Efficacious: sleep improves, TST↑<3h		
	Inefficacious: no improvement, or even worse		
**Method 2**	***Significant efficacious***: 30min > SOL, TST↑≥ 2h, PSQI global scores ≤ 7	Liu and Nan, 2009; Li and Gong, 2012; Gan et al., 2013; Wang X. et al., 2017; Wu and Jiang，2020	5 (27.8)
	Efficacious: 45min ≥ SOL ≥ 30min, TST↑≥ 1h, PSQI decline rate > 30%		
	Inefficacious: SOL > 45min, TST↑< 1h, PSQI decline rate < 25%		
**Method 3**	***Healed***: PSQI decline rate ≥ 95%	Chen et al., 2014; Yan et al., 2018; Kang, 2019	3 (16.6)
	Significant efficacious: 95% > PSQI decline rate ≥ 70%		
	Efficacious: 70% > PSQI decline rate ≥ 30%		
	Inefficacious: PSQI decline rate < 30%		
**Method 4**	***Healed***: PSQI decline rate ≥ 75%	Hu and Sheng, 2015; Wang J et al., 2017	2 (11.1)
	Significant efficacious: 75% > PSQI decline rate ≥ 50%		
	Efficacious: 50% > PSQI decline rate ≥ 25%		
	Inefficacious: PSQI decline rate < 25%		
**Method 5**	***Healed***: ISI decline rate ≥ 75%	Zhu et al., 2022	1 (5.6)
	Significant efficacious: 75% > ISI decline rate ≥ 50%		
	Efficacious: 50% > ISI decline rate ≥ 25%		
	Inefficacious: ISI decline rate < 25%		
**Method 6**	***Significant efficacious***: 30min ≥ SOL, TST ≥ 7h	Zhong, 2018	1 (5.6)
	Efficacious: 60min ≥ SOL, 6h ≥ TST ≥ 4h		
	Inefficacious: 4h > TST		

Abbreviations GCTNPCM, Guideline for Clinical Trials of New Patent Chinese Medicines; ISI, Insomnia Severity Index; PSQI, Pittsburgh Sleep Quality Index; SOL, sleep onset latency; TST, total sleep time.

Notes ISI decline rate = (pre-post ISI global scores difference)/pre-ISI global scores × 100%; PSQI decline rate = (pre-post PSQI global scores difference)/pre-PSQI global scores × 100%.

Appendix 5 Incidence of adverse events associated with each intervention

Adverse events	Involved studies	Incidence in different interventions		
		ZRAS (%)	Hypnotic (%)	ZRAS + Hypnotic (%)
dizziness	Li and Gong, 2012; Gan et al., 2013; Chen et al., 2014; Hu and Sheng, 2015; Liang, 2016; Wang X. et al., 2017; Yan et al., 2018; Yan et al., 2019; Wu and Jiang, 2020; Liu and Gong, 2021; Liu, 2022; Xu and Zhou, 2022; Ye and Lin, 2022; Zhu et al., 2022; Yang, 2023;	6/212 (2.8%)	56/499 (11.2%)	6/205 (2.9%)
dry mouth/bitter taste	Chen et al., 2014; Wang J et al., 2017; Wang X. et al., 2017; Yan et al., 2018; Cui, 2019; Yan et al., 2019; Liu and Gong, 2021; Birling et al., 2022; Liu, 2022; Ye and Lin, 2022; Zhu et al., 2022; Yang, 2023	5/201 (2.5%)	32/504 (6.3%)	3/113 (2.7%)
drowsiness (excessive daytime sleepiness)	Li and Gong, 2012; Gan et al., 2013; Chen et al., 2014; Hu and Sheng, 2015; Liang, 2016; Wang X. et al., 2017; Yan et al., 2018; Yan et al., 2019; Wu and Jiang, 2020; Liu, 2022; Ye and Lin, 2022; Zhu et al., 2022	4/160 (2.5%)	51/527 (9.7%)	2/71 (2.8%)
gastrointestinal symptoms (e.g., acid reflux, nausea, diarrhea, constipation, abdominal pain, etc.)	Gan et al., 2013; Chen et al., 2014; Hu and Sheng, 2015; Wang J et al., 2017; Cui, 2019; Wu and Jiang, 2020; Liu and Gong, 2021; Xu and Zhou, 2022; Ye and Lin, 2022; Zhu et al., 2022; Yang, 2023	11/374 (2.9%)	9/212 (4.2%)	4/141 (2.8%)
fatigue	Li and Gong, 2012; Gan et al., 2013; Hu and Sheng, 2015; Liang, 2016; Wang X. et al., 2017; Yan et al., 2018; Yan et al., 2019; Wu and Jiang, 2020; Liu, 2022; Xu and Zhou, 2022	18/291 (6.2%)	43/308 (14.0%)	11/232 (4.7%)
headache/head painful distension	Chen et al., 2014; Wang J et al., 2017; Cui, 2019; Xu and Zhou, 2022; Zhu et al., 2022	Ø	8/254 (3.1%)	1/48 (2.1%)

urinary urgency	Birling et al., 2022; Zhu et al., 2022	1/38 (2.6%)	1/35 (2.9%)	Ø
hangover effect	Li and Gong, 2012; Gan et al., 2013	Ø	5/90 (5.6%)	Ø
muscle soreness	Wu and Jiang, 2020; Liu and Gong, 2021	2/172 (1.2%)	1/52 (1.9%)	Ø
mouth-numbing	Chen et al., 2014; Wang J et al., 2017	2/112 (1.8%)	1/64 (1.6%)	Ø
frequent night waking	Birling et al., 2022	1/38 (2.6%)	Ø	Ø
facial skin rash	Birling et al., 2022	1/38 (2.6%)	Ø	Ø
sweat	Cui, 2019	1/43 (2.3%)	3/47 (6.4%)	Ø

Abbreviations ZRAS, Zao Ren An Shen.

Notes "Ø" for where the adverse events were not measured.

Appendix 6 Trial sequential analysis of PSQI global scores (ZRAS *Vs.* Hypnotic)

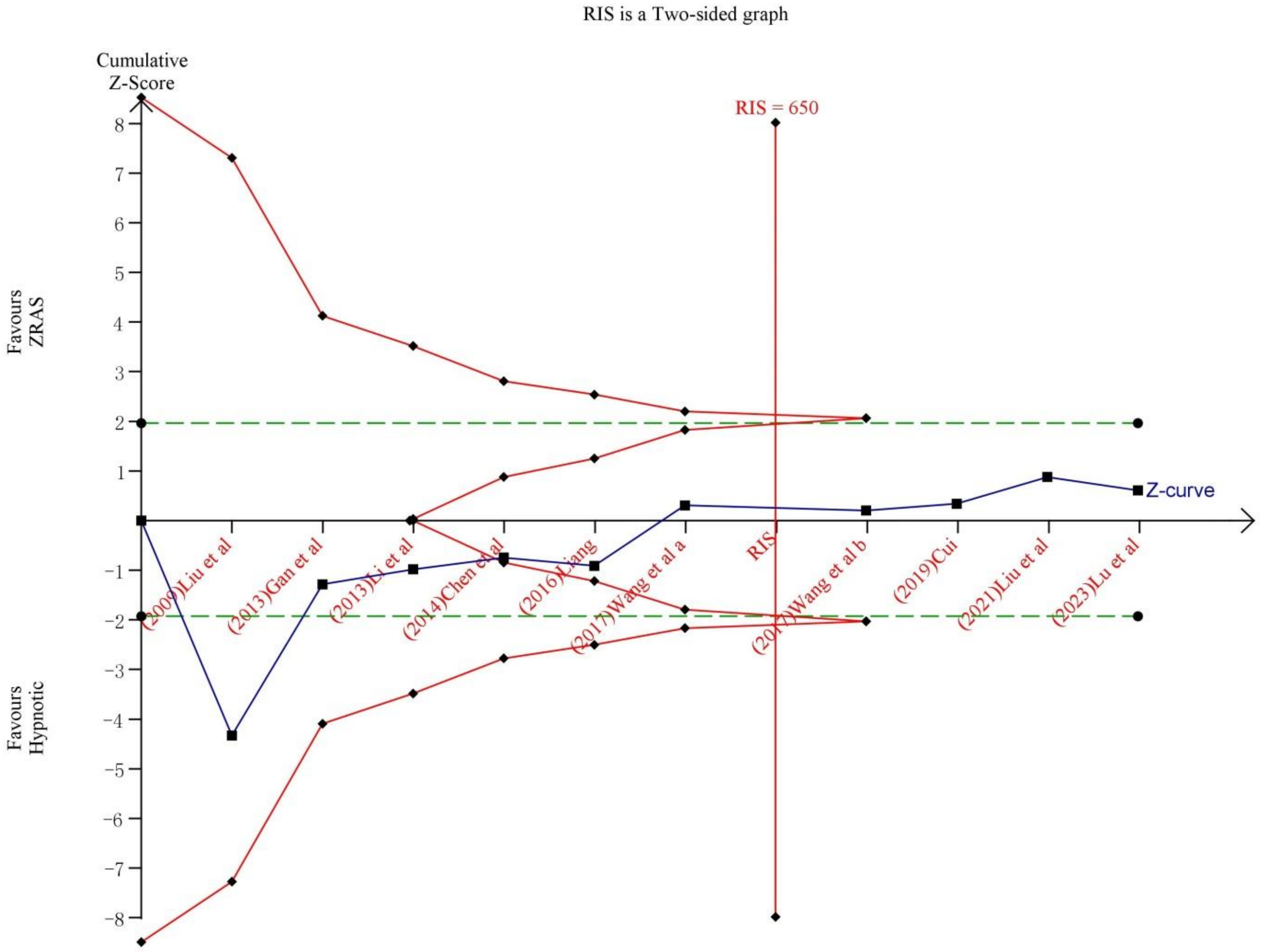

Appendix 7 Subgroup analyses of PSQI global scores (ZRAS *Vs*. Hypnotic)

Basis for subgroup classification	All trials or subgroup title	No. of Studies	No. of participants	Statistical method	Effect size	*p*	I^2	Subgroup analysis results
PSQI	All trials	10	871	Mean Difference (IV, Random, 95%*CI*)	-0.36 [-1.53, 0.81]	0.55	98	
Dosage form	(i) capsule	9	751	Mean Difference (IV, Random, 95%*CI*)	-0.36 [-1.61, 0.89]	0.57	98	Chi^2 statistic 0.00, *df* = 1, *p* = 0.99
	(ii) granule	1	120	Mean Difference (IV, Random, 95%*CI*)	-0.37 [-1.41, 0.67]	0.49	N/A	
Hypnotic used in the controls	(i) benzodiazepine	5	402	Mean Difference (IV, Random, 95%*CI*)	0.39 [-0.12, 0.91]	0.13	71	Chi^2 statistic 7.98, *df* = 1, *p* < 0.01
	(ii) Z-drug	5	469	Mean Difference (IV, Random, 95%*CI*)	-1.31 [-2.37, -0.24]	0.02	91	
Therapeutic dosage	(i) high (≥ 4 weeks)	7	669	Mean Difference (IV, Random, 95%*CI*)	-0.78 [-2.01, 0.46]	0.22	97	Chi^2 statistic 2.80, *df* = 1, *p* = 0.09
	(ii) low (< 4 weeks)	3	202	Mean Difference (IV, Random, 95%*CI*)	0.54 [-0.38, 1.46]	0.25	83	

Notes **Figure 3** demonstrates that ZRAS and hypnotic are equally effective in reducing PSQI scores, yet significant heterogeneity exists in the results. Therefore, subgroup analysis was conducted based on the dosage forms of ZRAS, hypnotic used in the controls, and therapeutic dosage.

Abbreviations PSQI, Pittsburgh Sleep Quality Index; N/A, Not applicable.

Appendix 8 Meta-regression analysis of PSQI global scores (ZRAS *Vs*. Hypnotic)

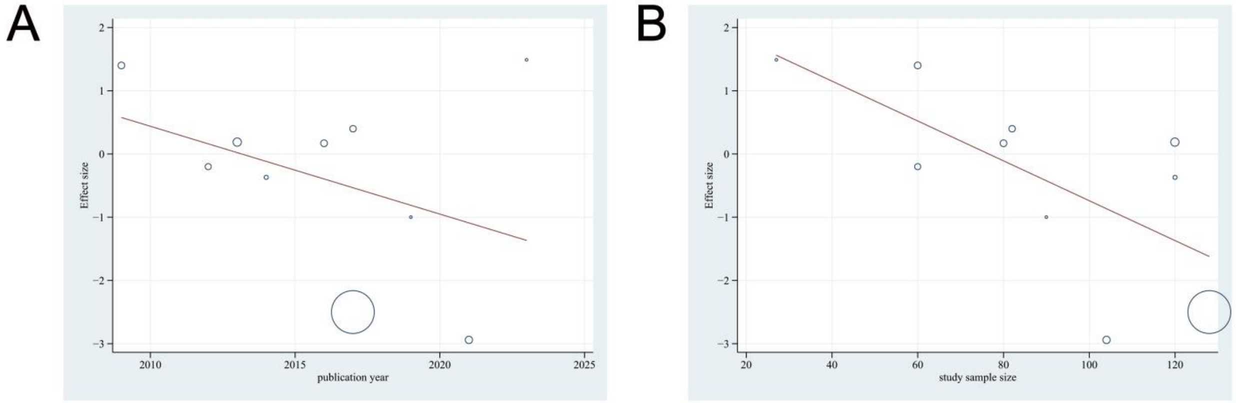

Notes Univariate meta-regression based on (A) publication year, (B) study sample size, (C) dosage forms of ZRAS, (D) hypnotics in the controls, and (E) therapeutic dosage.

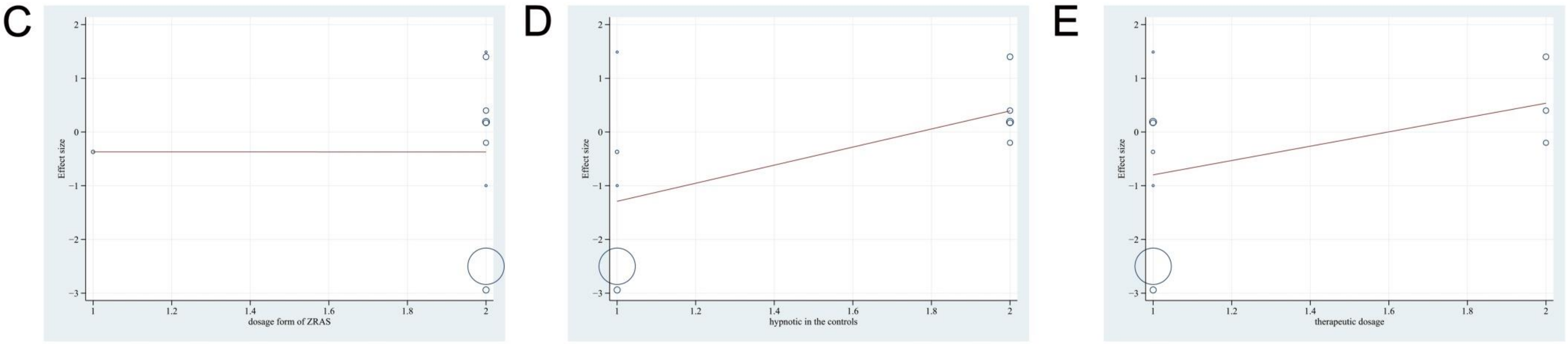

Appendix 9 Sensitivity analysis of PSQI global scores (ZRAS *Vs*. Hypnotic)

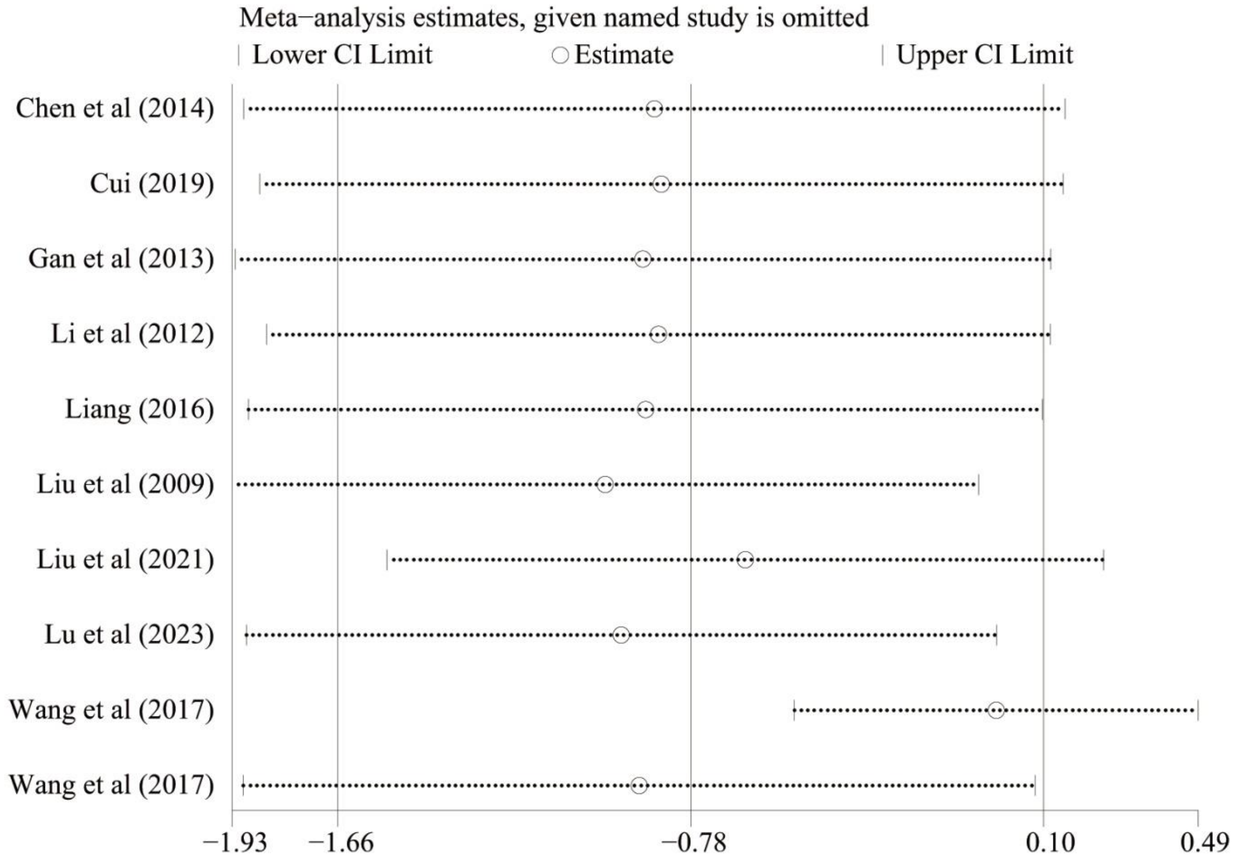

Appendix 10 Trial sequential analysis of PSQI global scores (ZRAS + Hypnotic *Vs.* Hypnotic)

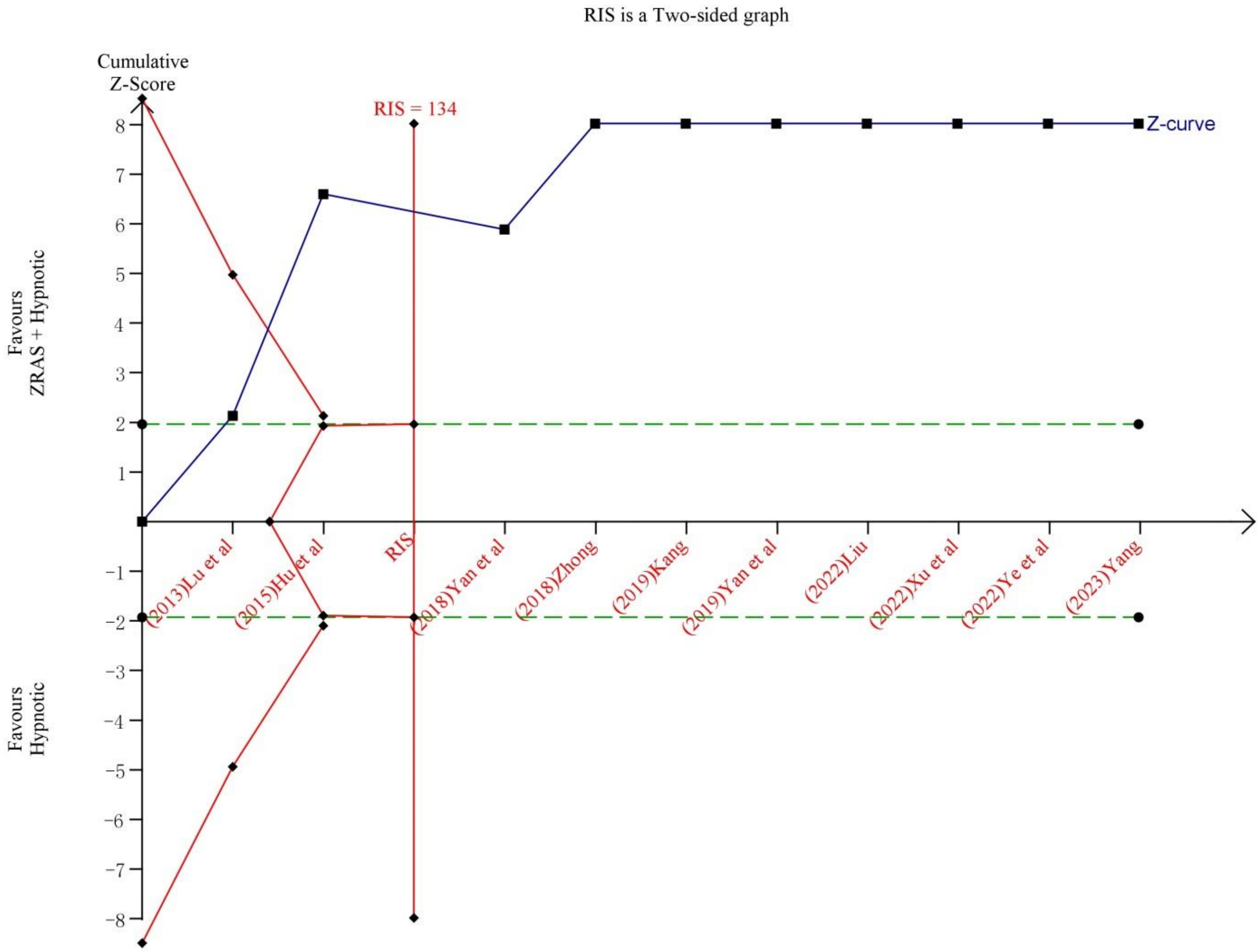

Appendix 11 Subgroup analyses of PSQI global scores, and polysomnography-recorded total sleep time and sleep onset latency (ZRAS + Hypnotic *Vs*. Hypnotic)

Basis for subgroup classification	All trials or subgroup title	No. of Studies	No. of participants	Statistical method	Effect size	*p*	I^2	Subgroup analysis results
PSQI	All trials	10	817	Mean Difference (IV, Random, 95%*CI*)	-2.70 [-3.22, -2.18]	< 0.01	88	
Study types	(i) RCT	5	353	Mean Difference (IV, Random, 95%*CI*)	-2.17 [-2.50, -1.83]	< 0.01	0	*Chi²* statistic 8.30, *df* = 1, *p* < 0.01
	(ii) CCT	5	464	Mean Difference (IV, Random, 95%*CI*)	-3.12 [-3.68, -2.57]	< 0.01	85	
Dosage form	(i) capsule	6	466	Mean Difference (IV, Random, 95%*CI*)	-2.95 [-3.56, -2.33]	< 0.01	87	*Chi²* statistic 3.48, *df* = 1, *p* = 0.06
	(ii) granule	4	351	Mean Difference (IV, Random, 95%*CI*)	-2.27 [-2.62, -1.91]	< 0.01	0	
Hypnotic used in the controls	(i) benzodiazepine	6	507	Mean Difference (IV, Random, 95%*CI*)	-2.64 [-3.18, -2.11]	< 0.01	77	*Chi²* statistic 0.02, *df* = 1, *p* = 0.89
	(ii) Z-drug	4	310	Mean Difference (IV, Random, 95%*CI*)	-2.72 [-3.73, -1.72]	< 0.01	86	
Therapeutic dosage	(i) high (≥ 4 weeks)	9	739	Mean Difference (IV, Random, 95%*CI*)	-2.77 [-3.31, -2.23]	< 0.01	87	*Chi²* statistic 2.23, *df* = 1, *p* = 0.14
	(ii) low (< 4 weeks)	1	78	Mean Difference (IV, Random, 95%*CI*)	-2.15 [-2.76, -1.54]	< 0.01	N/A	
Total sleep time	All trials	4	318	Mean Difference (IV, Random, 95%*CI*)	40.72 [25.14, 56.30]	< 0.01	56	
Study types	(i) RCT	1	60	Mean Difference (IV, Random, 95%*CI*)	47.40 [27.51, 67.29]	< 0.01	N/A	*Chi²* statistic 0.33, *df* = 1, *p* = 0.57
	(ii) CCT	3	258	Mean Difference (IV, Random, 95%*CI*)	39.21 [19.32, 59.10]	< 0.01	53	

Sleep onset latency	All trials	4	318	Mean Difference (IV, Random, 95%*CI*)	-4.44 [-7.98, -0.91]	0.01	96	
Study types	(i) RCT	1	60	Mean Difference (IV, Random, 95%*CI*)	-3.21 [-4.18, -2.24]	< 0.01	N/A	Chi^2 statistic 0.37, *df* = 1, *p* = 0.54
	(ii) CCT	3	258	Mean Difference (IV, Random, 95%*CI*)	-4.86 [-10.09, 0.38]	0.07	97	

Notes **Figure 4** illustrates that ZRAS and hypnotic are equally effective in reducing PSQI scores, yet significant heterogeneity exists in the results. Therefore, subgroup analysis was performed based on study types, dosage forms of ZRAS, hypnotic used in the controls, and therapeutic dosage.

Abbreviations PSQI, Pittsburgh Sleep Quality Index; CCTs, controlled clinical trials; RCTs, randomized controlled trials; N/A, Not applicable.

Appendix 12 Meta-regression analysis of PSQI global scores (ZRAS + Hypnotic *Vs*. Hypnotic)

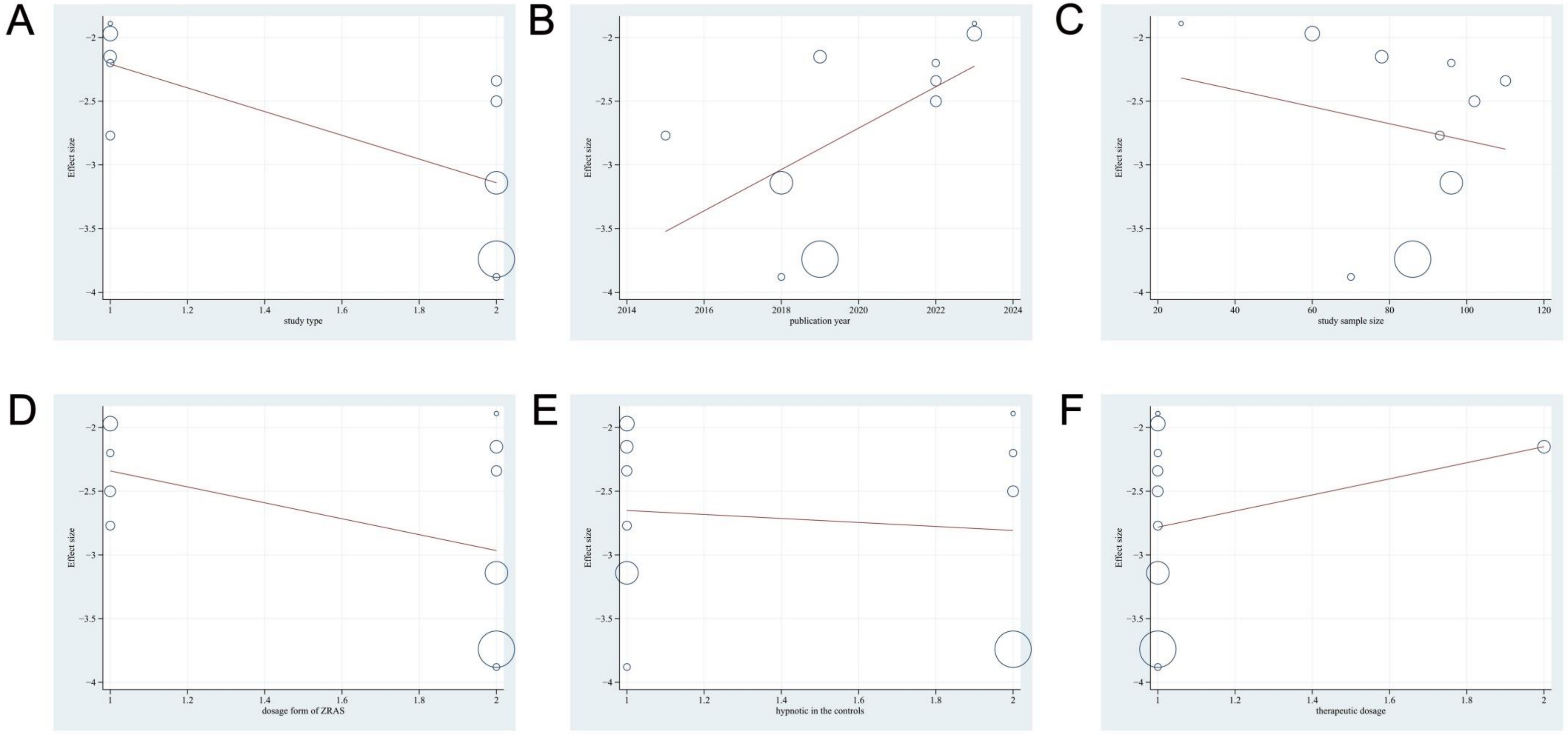

Notes Univariate meta-regression based on (A) study type, (B) publication year, (C) study sample size, (D) dosage forms of ZRAS, (E) hypnotics in the controls, and (F) therapeutic dosage.

Appendix 13 Sensitivity analysis of PSQI global scores (ZRAS + Hypnotic *Vs*. Hypnotic)

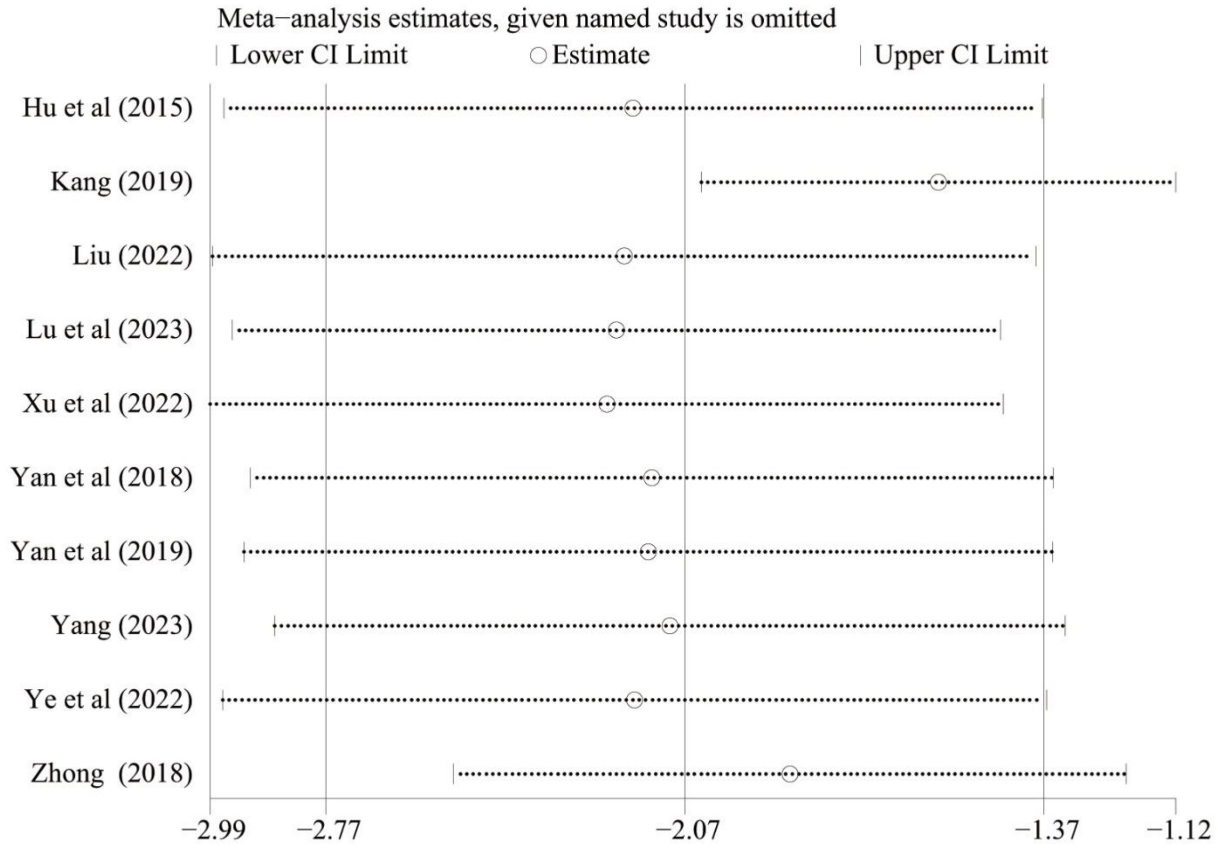

Appendix 14 Publication bias test based on PSQI global scores

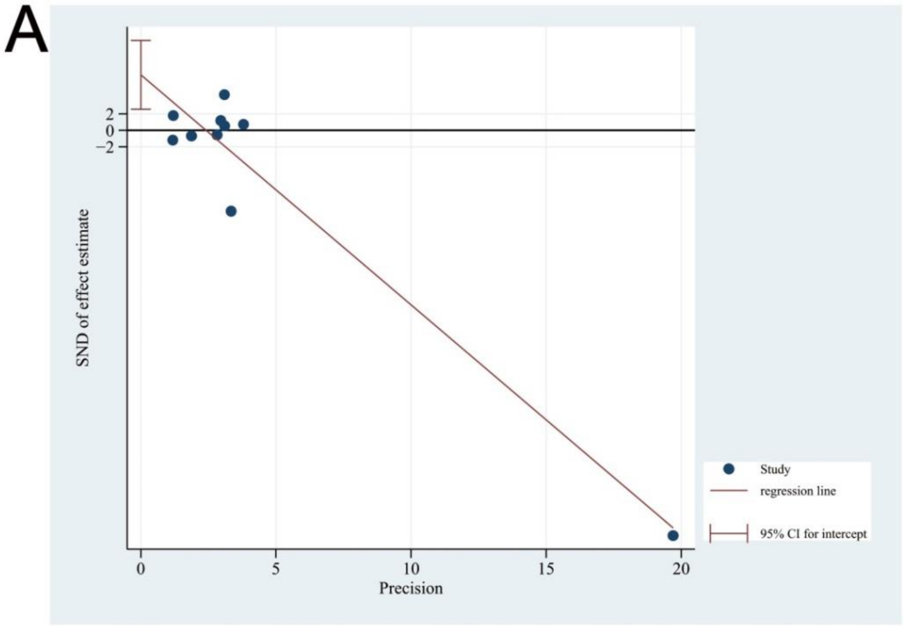

(A) Publication bias in ZRAS *Vs.* Hypnotic.

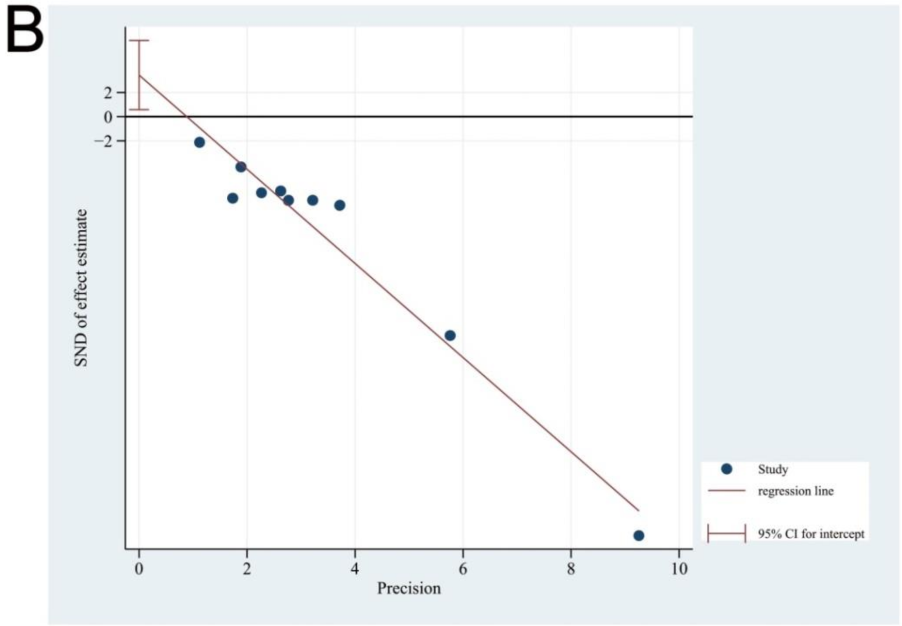

(B) Publication bias in ZRAS + Hypnotic *Vs.* Hypnotic.

Appendix 15 Evidence quality assessment of major outcome measures based on GRADE system

Interventions *Vs*. Controls	Outcomes	Numbers of studies (numbers of participants)	Estimated effects (MD/RR with 95%*CI*)	I^2% (*p*)	Initial grade for strength of evidence	Limitations	Inconsistency	Indirectness	Imprecision	Publication bias	Certainty of evidence
ZRAS *Vs*. Hypnotic	PSQI global scores	10 (871)	MD = -0.36 [-1.53, 0.81]	98 (0.55)	High	-1①	-1②	0	-1③	-1④	Very low
	Clinical efficacy rate	8 (717)	RR = 1.05 [0.96, 1.15]	48 (0.31)	High	-1①	0	0	-1③	0	Low
ZRAS + Hypnotic *Vs*. Hypnotic	PSQI global scores	10 (817)	MD = -2.70 [-3.22, -2.18]	88 (< 0.01)	Low	-1①	-1②	0	0	-1④	Very low
	Total sleep time	4 (318)	MD = 40.72 [25.14, 56.30]	56 (< 0.01)	Low	-1①	-1②	0	-1③	0	Very low
	Sleep onset latency	4 (318)	MD = -4.44 [-7.98, -0.91]	96 (0.01)	Low	-1①	-1②	0	-1③	0	Very low
	Number of awakenings	3 (232)	MD = -0.89 [-1.67, -0.10]	93 (0.03)	Low	-1①	-1②	0	-1③	0	Very low
	Clinical efficacy rate	10 (858)	RR = 1.23 [1.16, 1.31]	0 (< 0.01)	Low	-1①	0	0	0	0	Very low

Abbreviations: PSQI, Pittsburgh Sleep Quality Index.

Notes GRADE [①The design of the trial has a large bias in randomization, allocation concealment, blinding or other factors; ②the confidence interval overlaps less, the heterogeneity test *p* is very small, the I^2 is larger, and the heterogeneity could not be completely explained by conducting subgroup analysis, sensitivity analysis, or meta-regression; ③the confidence interval is not narrow enough; ④funnel graph asymmetry]. The GRADE approach classifies bodies of randomized controlled trials as initially starting at high certainty and bodies of non-randomized studies at initially starting at low certainty.
